# Assessing the *In Vitro* Effects of Carrot Pomace Extract on Intestinal Epithelium Integrity and Functions

**DOI:** 10.3390/antiox15070847

**Published:** 2026-07-04

**Authors:** Ana Maria Ciupitu, Gina Cecilia Pistol, Valeria Cristina Bulgaru, Iulian Alexandru Grosu, Alexandra Gabriela Oancea, Norica Branza-Nichita, Ionelia Taranu

**Affiliations:** 1 Laboratory of Animal Biology, National Institute of Research and Development for Biology and Animal Nutrition, 077015 Balotesti, Romania; ana.ciupitu@ibna.ro (A.M.C.); cristina.bulgaru@ibna.ro (V.C.B.); grosu.iulian@ibna.ro (I.A.G.); alexandra.oancea@ibna.ro (A.G.O.); ionelia.taranu@ibna.ro (I.T.); 2 SCOSAAR-Advanced Studies School of the Romanian Academy, Institute of Biochemistry, 060031 Bucharest, Romania; nichita@biochim.ro; 3 Laboratory of Feed and Food Quality, National Institute of Research and Development for Biology and Animal Nutrition, 077015 Balotesti, Romania

**Keywords:** carrot pomace, polyphenols, antioxidants, bioactive compounds, IPEC1 cells, porcine intestinal epithelium, LPS, cellular proliferation, apoptosis, ERK1/2 MAPKs, inflammation, oxidative stress

## Abstract

Carrot processing for juice generates substantial pomace residues rich in bioactive compounds, which represent both an environmental challenge and an unexploited resource. This study investigated the protective effects of a polyphenolic extract derived from carrot pomace (CP) against *Escherichia coli* lipopolysaccharide (LPS)-induced damage. For that, we used IPEC-1 (Intestinal Porcine Epithelial Cells) as an *in vitro* model of the intestinal epithelium. The total phenolic content of the CP polyphenolic extract (CPE) was 1.017 mg GAE/mL, with flavan-3-ols (epicatechin, catechin, epigallocatechin) accounting for 71.3% of that value. Before being exposed to LPS (10 μg/mL) for 24 h, the cells were pre-treated with CP extract (20.34 µg and 10.17 µg polyphenols/mL of extract corresponding to 1/50 and 1/100 dilution) for 4 h. Epithelial renewal (cell viability, cell proliferation and apoptosis), monolayer/barrier integrity (TEER, FD4 permeability, LDH release), as well as epithelial functionality (synthesis of pro-inflammatory cytokines: TNF-α, IL-1β, IL-6, reactive oxygen species (ROS), nitric oxide (NO) production), MAPK signalling and mitochondrial morphology and function were assessed. The results showed that CP extract had no cytotoxic effects and successfully counteracted LPS-induced loss of cell viability and proliferation. The pre-treatment with CPE at both dilutions significantly reduced LPS-induced apoptosis and cell death. Barrier integrity was preserved with TEER values maintained near baseline: −0.43% and −0.24% for 1/50 and 1/100 dilutions of CPE *vs*. −53.47% at 72 h for LPS alone, and paracellular FD4 passage was restored to control levels. At the molecular level, CP extract reduced pro-inflammatory cytokine gene expression (*IL-6* by 40%, *TNF-α* by 50–56%) and suppressed LPS-induced MAPK activation by 62.9% and 46.5%, for 1/50 and 1/100 dilutions of CPE, respectively. The pre-treatment of cells with CP extract normalised LPS-induced ROS production and protected mitochondrial morphology and function. These *in vitro* findings demonstrate that CP extract exerts a protective effect on intestinal epithelial cells, acting through anti-inflammatory, antioxidant and barrier-preserving mechanisms. This supports the hypothesis for valorisation of carrot agro-industrial by-products as functional feed additives for promoting intestinal health. Further *in vivo* studies are needed to validate this hypothesis and to establish the concentration/rate of inclusion of carrot by-products to achieve the maximal positive effects.

## 1. Introduction

The fruit and vegetable sector represents one of the largest and fastest-growing segments of the global agricultural production market, offering a wide range of products, including juices, jams and dehydrated products [1]. This sector generates significant amounts of residues and by-products that are rich in nutrients with anti-inflammatory and antioxidant properties. Some studies showed that incorporating plant residues into animal feed enhances immune responses through their content of bioactive nutrients [2,3]. After the ban on antibiotics in 2006 and zinc oxide in 2023 for use in animal feed [4], the research sector has faced an urgent need to develop alternative solutions to replace antibiotics as growth promoters in animal production. This created challenges and, at the same time, opportunities for the animal nutrition industry, demanding innovative approaches to maintain animal health and productivity. Recent studies have demonstrated that residues from fruit and vegetable processing represent a promising alternative for improving immune status, intestinal health and overall performance in livestock [5,6]. These findings highlight the potential of valorising agricultural by-products as functional feed components with health-promoting properties.

The carrot (*Daucus carota*) is one of the most important root vegetables in the *Apiaceae* family, whose fleshy root is widely consumed worldwide [7]. Carrot roots are abundant in carotenoids, phenolic acids, anthocyanins (found especially in purple and black carrots), dietary fibre, and vitamins, all of which function as antioxidants and play a crucial role in immunological response, cardiovascular function, digestive health and cancer prevention [8]. Numerous factors, including growing techniques, fertilisers and environmental conditions, influenced the mineral and phytochemical composition of carrots [9].

The juice industry generates thousands of tons of pomace annually, with 30 to 50% of the carrots used for juice production remaining as residues [10]. This substantial amount of pomace represents an environmental challenge and an unexploited resource for valuable bioactive compounds. CP contains uronic acids, neutral sugars, carotenoids, dietary fibre, and polyphenols, but especially phenolic acids [11]. Up to 50% of the total carotenoid content of carrots is retained in the pomace [11], suggesting that a significant portion of the nutritional value is preserved in this by-product.

To evaluate the biological effects of carrot residue extract, this study employed the intestinal porcine cell line, IPEC-1 primary cells from pig as an *in vitro* model system. They are investigating how intestinal epithelial cells interact with CP extract is particularly relevant, as these cells are in direct contact with the digesta, express transporters and proteins involved in nutrient absorption and intestinal barrier function serving also as the first line of defence against pathogens and toxins. Cultivated epithelial cell lines such as IPEC-1 (Intestinal Porcine Epithelial Cells) serve as crucial *in vitro* resources for such investigations, offering a controlled and reproducible system to study cellular responses to dietary compounds.

The gastrointestinal epithelium functions as a critical, dynamic interface that mediates essential nutrient absorption while simultaneously acting as a highly selective physical and immunological barrier [12]. This dual function requires a balance between permeability for nutrient uptake and barrier integrity to prevent pathogen invasion. Maintenance of this barrier integrity is fundamental to overall health, especially in monogastric animals such as swine, which frequently face environmental stressors and pathogenic challenges, particularly during the weaning period [13].

Animal studies have demonstrated significant antioxidant and hepatoprotective properties of carrot extracts, as evidenced by the reduction of oxidative stress, enhancement of biochemical hepatic markers and histological preservation of affected tissues, particularly in scenarios of toxicity or experimentally induced diabetes [14]. *In vitro* studies have shown that carrot extracts reduce inflammation and oxidative stress, regulate glucose metabolism, and inhibit cancer cell growth in both animal and human cell lines [15,16]. These studies underlined the effects of the carrot pomace extracts on the metabolic and intestinal cellular parameters, as well as the molecular mechanisms involved. Recent studies have demonstrated that incorporating carrot by-products such as fermented CP in the pig’s diet can enhance growth rates, improve gut microbiota health, increase antioxidant capacity, while maintaining the meat quality [17,18]. Given the physiological and metabolic similarities between pigs and humans [19], as well as the importance of this model in nutrition and digestive physiology, the investigation of CP effects in porcine models is justified and may provide valuable information for both zootechnical applications and potential biomedical uses [20].

In this context, the objective of this study was to investigate the ability of a total polyphenolic extract derived from carrot pomace to counteract the effects of *Escherichia coli* endotoxin lipopolysaccharide (LPS) on the intestinal epithelium.

## 2. Materials and Methods

### 2.1. Characterisation of Carrot Pomace and Carrot Pomace Extract

#### 2.1.1. Obtaining the Carrot Pomace and the Carrot Pomace Extract

Common orange carrots (*Daucus carota sativum*) were purchased from a local commercial farmer, and after juice extraction, the residue was dried and ground using a lyophilizer (Telstar LyoQuest, Bench Top Laboratory Freeze-dryer, © Syntegon Telstar, SLU Barcelona, Spain).

To obtain the extract from the CP residue, 1 g of carrot residue was mixed with 7 mL of 80% methanol. The tube containing the resulting mixture was placed in a shaker at 250 rotations per minute (min), at 37 °C, for 24 h (h), then centrifuged at 3000 rpm for 15 min. After centrifugation, the sample was separated into two phases: solid and liquid. The liquid phase was distributed into several tubes and introduced into the extractor (Christ, RVC 2-18 CD plus, Martin Christ Gefriertrocknungsanlagen GmbH, Osterode am Harz, Germany) for methanol evaporation according to the evaporation program. After evaporating the methanol, we obtained 1.4 mL of aqueous extract from the carrot residue containing 1424 μg of polyphenols. The obtained extract was stored at −20 °C until subsequent analyses. The extraction was performed in three replicates.

#### 2.1.2. Detection of Total Polyphenols and Antioxidant Activity

The concentration of total polyphenols was assessed using the Folin–Ciocalteu technique modified for microscale use, as described by Vlassa et al. [21]. The absorbance was measured at 750 nm using a plate reader (Tecan, Infinite M200 Pro, Tecan Trading AG, Männedorf, Switzerland), and the total polyphenol content of the CP extract was determined using a gallic acid standard calibration curve. The results were presented as gallic acid equivalents (mgGAE). The antioxidant activity was measured using the DPPH (2,2′-diphenyl-1-picrylhydrazyl radical) assay as described by Vlassa et al. [21]. The samples were prepared as analytical duplicates. The scavenging activity of DPPH was calculated using the following equation:

DPPH scavenging activity (%) = {(Abs control − Abs sample)/Abs control} × 100

The effective concentrations were expressed in µmol Trolox equivalents/100 g of dry matter.

#### 2.1.3. Measurement of Polyphenol Composition by High-Performance Liquid Chromatography-Photodiode Detector Coupled with Mass Spectroscopy (HPLC-DAD-MS)

Phenolic compounds have been identified and characterised by using the liquid chromatography technique described by Untea et al. [22]. The measurement was performed by using a Vanquish Core HPLC system equipped with a DAD from Thermo Fisher Scientific located in Bremen, Germany. The system used a BDS HyperSil C18 column with dimensions of 250 × 4 mm and a particle size of 5 µm. A binary gradient was implemented, consisting of 1% acetic acid in distilled water as solvent A, methanol as solvent B, and acetonitrile as solvent C, at a flow rate of 0.5 mL/min. The elution protocol was classified into specific time intervals: from 0 to 15 min, 5% solvent B and 5% solvent C were used. From 15 to 20 min, 4% solvent B and 15% solvent C were used; from 20 to 25 min, 3% solvent B and 25% solvent C were used; from 25 to 40 min, 2% solvent B and 38% solvent C were used; and from 40 to 50 min, the solution reverted to 5% solvent B and 5% solvent C. The identification of phenolic compounds was completed by comparing sample peaks with individual reference standards.

### 2.2. Experimental Design

#### 2.2.1. IPEC-1 Cells Culture

The IPEC-1 (Intestinal Porcine Epithelial Cell line-1), kindly provided by Dr. P. Pinton from INRAe, France, is a non-transformed cell line derived from the small intestine (jejunum-ileum tissue) obtained from unweaned pigs under 12 h of age. Due to its capacity to replicate the intestinal barrier, the IPEC-1 cell line is mostly used for investigating epithelial transport, bacterial interactions, the impact of microorganisms, and the impact of various compounds that may be useful or toxic to gut health.

#### 2.2.2. Inflammatory and Bacterial Challenge with LPS-*Escherichia coli*

Lipopolysaccharides (LPS) are vital components of the cell wall in Gram-negative bacteria like *Escherichia coli*, consisting of lipid A, a short core oligosaccharide, and a distal polysaccharide (O-antigen). The LPS used was derived from *E. coli* serotype O26:B6 (Sigma-Aldrich, St. Louis, MO, USA), which is known for its strong capacity to activate TLR4 and to induce inflammation and oxidative stress.

#### 2.2.3. Cell Treatments

IPEC-1 cells were defrosted, washed with full medium, and cultivated in T75 flasks. Upon achieving confluence, the cells were detached from the culture vessel wall using trypsin-EDTA solution (Sigma-Aldrich, St. Louis, MO, USA). The isolated cells were counted using a Neubauer counting chamber and then cultured in 96-, 24- or 6-well plates (Corning, Sigma-Aldrich, St. Louis, MO, USA) at a density of 1 × 10^5^ cells/mL. The cultivation was performed at 37 °C in an environment supplemented with 5% CO_2_. The complete culture medium was formulated utilizing DMEM/F12 medium (Sigma) enhanced with 5% fetal bovine serum (Sigma-Aldrich, St. Louis, MO, USA), 1% penicillin/streptomycin (10,000 units of penicillin/mL and 10 mg streptomycin/mL), 1% L-glutamine (Gibco BRL, Thermo Fisher Scientific, Waltham, MA, USA), 1% ITS (Insulin/Transferrin/Selenium Gibco BRL, Thermo Fisher Scientific, Waltham, MA, USA), and 0.05% Epidermal Growth Factor (EGF–LifeSpan BioScience, Newark, CA, USA), and was renewed every 2–3 days until cells confluence was achieved.

Cellular viability and treatment cytotoxicity were assessed in IPEC-1 cells using the MTT assay. The IPEC-1 cells were seeded at a concentration of 1 × 10^5^ cells/mL in complete medium until reaching 80% confluence (2 days). Then, the cells were treated with CP extract (dilutions of 1/50 and 1/100) for 4 h and challenged with *E. coli* LPS for another 24 h. Cell viability was assessed using the MTT [3-(4,5-dimethylthiazol-2-yl)-2,5-diphenyl tetrazolium bromide] assay, according to the manufacturer’s instructions. After the incubation period, the absorbance was measured at 450 nm using an ELISA microplate reader (Tecan Infinite M200 PRO, Vienna, Austria). Cellular viability was expressed as a percentage of the control, which is considered 100%. Three independent experiments (three independent biological replicates) were performed.

To investigate the effect of CP extract on the IPEC-1 cell line, we challenged the cells with 10 μg/mL of *E. coli* lipopolysaccharide (LPS, Sigma-Aldrich, St. Louis, MO, USA), used as an inducer of inflammation and oxidative stress often encountered in the intestine during the weaning period, for example. Six experimental conditions were tested:

(1) Control: untreated cells,
(2) Treatment with CP extract (1): cells treated with CP extract alone (equivalent to 20.34 µg polyphenols/mL of extract, 1/50 dilution),
(3) Treatment with CP extract (2): cells treated with CP extract alone (equivalent to 10.17 µg polyphenols/mL of extract, 1/100 dilution),
(4) Treatment with LPS (cells challenged with LPS at 10 μg/mL),
(5) Cells treated with CP extract (1) for 4 h and then challenged with LPS,
(6) Cells treated with CP extract (2) for 4 h and then challenged with LPS.

The two concentrations were expressed as dilutions further in the article.

### 2.3. Analytical Methods

#### 2.3.1. Cell Proliferation and Apoptosis Assessment

- Cell proliferation measurement

The IPEC-1 cells were seeded at a concentration of 1 × 10^5^ cells/mL in complete culture medium and maintained in 6-well plates until reaching 80% confluence (2 days). Then, the culture was treated with CP extract (dilution 1/50 and 1/100) for 4 h and challenged with *E. coli* LPS (10 μg/mL) for another 24 h. Cell proliferation was measured as Ki67 expression using the Muse^®^ Ki67 Proliferation Kit (Luminex Corporation, Austin, TX, USA), according to the manufacturer’s recommendations. Briefly, cells were harvested, washed with PBS, fixed, and then permeabilised via incubation with permeabilisation buffer for 15 min at room temperature. Then, the cells were stained with 10 μL of either Muse^®^ Hu IgG1-PE or Muse^®^ Hu Ki67-PE, incubated for 30 min at room temperature, and the percentages of Ki67-positive cells in the control and treated cells were measured using the Guava Muse™ Cell Analyser (Luminex Corporation, Austin, TX, USA). Three independent experiments (three independent biological replicates) were performed.

- Apoptosis measurement

Apoptosis was measured by flow cytometry using the Muse^®^ Annexin V & Dead Cell Kit (Luminex Corporation, Austin, Texas, USA) and the Guava^®^ Muse^®^ Cell Analyser for acquisition of live, early-apoptotic, late-apoptotic, and dead cells. The cytometry analysis was performed on IPEC-1 cells after they reached 80% confluence. The cells were treated with CP extract at dilutions of 1/50 and 1/100 for 4 h. Subsequently, they were challenged with *E. coli* LPS at 10 μg/mL for 24 h. For this analysis, 100 μL of Muse^®^ Annexin V and Dead Cell Reagent was mixed with 100 μL of cell suspension, incubated for 20 min at room temperature and then read on the Guava Muse Cell Analyser, which provides percentages of live, early apoptotic, late apoptotic, total apoptotic, and dead cells. Three independent experiments (three independent biological replicates) were performed.

#### 2.3.2. Intestinal Epithelial Integrity and Inflammatory Response Assessment

- Monolayer damage detection measurement (LDH Assay)

The activity of lactate dehydrogenase (LDH) in the IPEC-1 culture was measured using a commercial kit (Sigma-Aldrich, St. Louis, MO, USA). The cells were treated with CP extract dilutions of 1/50 and 1/100 for 4 h, followed by exposure to *E. coli* LPS at 10 μg/mL for an additional 24 h. The same volume (25 μL) of cells and assay buffer was combined in a 96-well plate, followed by the addition of 50 μL of substrate mix diluted in assay buffer. Additionally, the plate was incubated at 37 °C, and absorbance was measured every 30 s until the maximum absorbance of the samples surpassed the highest standard value (12.5 nmol/well). LDH activity was determined according to the kit’s instructions. Absorbance measurements were conducted after 2–3 min using a Varioskan™ LUX multimode microplate reader (Thermo Fisher Scientific, Waltham, MA, USA). Three independent experiments (three independent biological replicates) were performed.

- Trans-Epithelial Electrical Resistance Measurement (TEER)

Cells were seeded at a density of 1 × 10^5^ cells/mL in 24-well clear PET membrane inserts (Falcon, Corning, Durham, NC, USA) with 0.4 μm holes and grown in complete medium. The cell media was changed every two days until full differentiation occurred. The cells were then treated with CP extract at 1/50 and 1/100 dilutions for 4 h and challenged with 10 μg/mL *E. coli* LPS for an additional 24 h. The TEER was measured at 0, 24, 48, and 72 h using a Millicell ERS-2 Voltohmmeter (Millipore, Merck, Darmstadt, Germany). The experimental TEER values were given as kOhms × cm^2^. Three independent experiments (three independent biological replicates) were performed.

- Measurement of the cellular permeability

Cells were seeded at 1 × 10^5^ cells/mL in 24-well plates with 0.4 μm membrane inserts and cultured in complete DMEM/F12 medium until they were fully differentiated. After differentiation, the cells were treated with CP extract at 1/50 and 1/100 dilutions for 4 h and challenged with 10 μg/mL *E. coli* LPS for 24 h. The transport of fluorescein isothiocyanate (FITC)-conjugated dextran 4 kDa (FD4) dissolved in PBS to a final concentration of 1 mg/mL was used to assess the paracellular permeability of the cellular monolayers. The transwells containing the treated or untreated cells were incubated for 2 h at 37 °C in the dark, after addition of 100 μL of FD4 solution to the apical compartment. Samples of the supernatant from the apical and basal compartments of the transwell systems were collected to quantify the passage of FD4 across the monolayer. A Varioskan^TM^ LUX multimode microplate reader (Thermo Fisher Scientific, Waltham, MA, USA) was then used to measure FD4 fluorescence at 490 nm excitation and 520 nm emission. Using a calibration curve, FD4 concentration in samples was expressed as μg/mL. Three independent experiments (three independent biological replicates) were performed.

- Assessment of pro-inflammatory cytokine gene expression

The real-time PCR (qPCR) method was used to measure the expression of genes coding for several molecules involved in the inflammatory response and oxidative stress. After the treatments, the cells were collected and homogenised in 700 µL QIAzol^®^ Lysis Reagent (QIAGEN, GmbH, Hilden, Germany). Following the manufacturer’s instructions, the RNeasy Mini kit (QIAGEN, GmbH, Hilden, Germany) was used to extract the total RNA. The amount and quality of isolated RNA were checked using the Agilent 4150 TapeStation spectrophotometer (Agilent Technologies, Santa Clara, CA, USA). Next, the M-MLV Reverse Transcriptase Kit (Life Technologies, Carlsbad, CA, USA) was used to reverse-transcribe RNA into complementary DNA (cDNA), following the manufacturer’s instructions. The qPCR was performed using the Rotor-GeneQ system (QIAGEN GmbH), and gene expression of pro-inflammatory cytokines (*TNF-α*, *IL1β* and *IL-6*) was assessed. The sequences of primers used are presented in Supplementary Table S1. Two reference genes (*ACTB*: β-actin and *RPL32*: Ribosomal Protein L32) were selected from a panel of six reference genes, using NormFinder Excel-based software [23] and were used for the normalisation of qPCR data. For the analysis of the qPCR data, the 2^(−ΔΔCT)^ method was used [24], and the obtained results were expressed as relative fold change (Fc) compared to untreated (Control) cells. Three independent experiments (three independent biological replicates) were performed.

- Assessment of signalling molecules involved in proliferation, inflammation, and apoptosis

To measure the ERK1/ERK2 MAPK (Mitogen-Activated Protein Kinases) expression by flow cytometry, we used the Muse^®^ MAPK Activation Dual Detection Kit. This is a two-colour kit featuring two conjugated antibodies that allow simultaneous detection of total and phosphorylated ERK levels in cells, leading to an accurate assessment of MAPK activation. The cells were treated with CP extract for 4 h after reaching confluence (3 days) and challenged with LPS at 10 µg/mL for 24 h. After incubation, the cells were detached, washed with PBS, centrifuged, resuspended in 500 µL of fixative solution and incubated on ice for 5 min. Cells were then permeabilised with 500 µL of solution for 5 min on ice, centrifuged, and the cell pellet was labelled with 10 µL of a working cocktail solution consisting of two directly conjugated antibodies: anti-phospho-ERK1/2(Thr202/Tyr185/Tyr187)-Phycoerythrin and an anti-ERL1/2-PECy5 conjugate. After incubation for 30 min at room temperature (protected from light), the cells were centrifuged, washed and resuspended in 200 µL of working buffer. The percentages of total and activated ERK1/ERK2 MAPK-labelled cells were determined by using the Guava Muse Cell Analyser. Three independent experiments (three independent biological replicates) were performed.

#### 2.3.3. Oxidative Stress Assessment

- Assessment of the Oxidative (ROS) and Nitrosative Stress (NO)

Oxidative stress (quantified by generation of Reactive oxygen species—ROS) and nitrosative stress (quantified by generation of Nitric Oxide—NO) were measured by flow cytometry using kits from Luminex Corporation, Austin, Texas, USA. Cells were cultured in 24-well plates at a density of 1 × 10^5^ cells/mL. After the cells reached confluence, the media was changed every two days. After differentiation, IPEC-1 cells were treated with CP extract at 1/50 and 1/100 dilutions for 4 h, then challenged with 10 μg/mL *E. coli* LPS and incubated for another 24 h. After removing the supernatant, the cells were detached, recovered in 0.5 mL PBS, and stained for ROS and NO expression. The Muse Oxidative Stress Kit (Luminex Corporation, Austin, Texas, USA) was used to assess the antioxidant effects of the CP extract on IPEC-1. ROS-expressing cells were quantified to assess oxidative stress in IPEC-1 cells. In summary, 10 µL of cells (1 × 10^5^ cells/mL) were mixed with 190 μL of Oxidative Stress Reagent and incubated for 30 min at 37 °C. The percentages of cell populations labelled ROS (+) or ROS (−) were used to express the results. The Muse Nitric Oxide Kit (Luminex Corporation, Austin, TX, USA) was used to assess the effects of the CP extract on IPEC-1 cells at nitrosative stress levels. For this analysis, 100 μL of Muse Nitric Oxide working solution was mixed with 10 μL of cells and incubated at 37 °C for 30 min. After incubation, 90 μL of Muse 7-AAD working solution was added to cells, mixed thoroughly, and then read on the Guava Muse Cell Analyser. The Muse^®^ Cell Analyser 1.5 software (version 1.5.0.0, developed by Merck Millipore, Darmstadt, Germany) was used to calculate the proportion and concentration of live cells, live cells with NO activity, dead cells with NO activity, dead cells, and total NO-positive cells (live and dead). Three independent experiments (three independent biological replicates) were performed.

#### 2.3.4. Mitochondrial Morphology and Function Assessment

- Assessment of Mitochondrial Morphology

The Mito-Lite Red FX600 (AAT Bioquest, Pleasanton, CA, USA), a collection of fluorogenic probes for labelling the mitochondria of live cells, was used to assess mitochondrial morphology and cell viability. The mitochondrial dyes accumulate in mitochondria via the mitochondrial membrane potential gradient. The IPEC-1 cells were seeded at a density of 1 × 10^5^ cells/mL of culture medium in 24-well microplates and grown until 100% confluence was reached (3 days). The culture was then exposed to CP extract (dilutions of 1/50 and 1/100) for 4 h and challenged with *E. coli* LPS (10 μg/mL) for 24 h. After the incubation, the culture medium was removed, and the cells were washed with PBS and treated with 2 μL of a 500 × MitoLite stock solution in 1000 μL of PBS for 2 h at 37 °C. The cells were then washed with warm PBS, and the fluorescence intensity was assessed using a Nikon ECLIPSE Ts2RFL fluorescence microscope (New York, NY, USA) equipped with a Cy5 HYQ red excitation filter. Three independent experiments (three independent biological replicates) were performed.

Mitochondrial network analysis: The images of the MitoLite Red FX600-stained cells were processed using ImageJ software (1.54r version), as described by Valente et al. [25]. The images were processed and skeletonised, and the MiNA plugin from ImageJ software was used to quantify the mitochondrial morphological parameters in IPEC1 cells.

- Assessment of mitochondrial function

The Muse MitoPotential kit (Luminex Corporation, Austin, Texas, USA) measures two crucial cell health indicators simultaneously: mitochondrial potential change, which is thought to be an early indicator of apoptosis, and cellular plasma membrane permeabilisation, a parameter of cell death. The cells were treated in a similar manner to the ROS and NO analysis. Muse MitoPotential working solution (95 μL) was added to 100 μL of cells, and incubated at 37 °C for 20 min. After the incubation, 5 μL of Muse 7-AAD was added to each sample and incubated at room temperature for 5 min. The cells were then mixed thoroughly and run on the Muse Cell Analyser. The percentages of live, depolarised, depolarised/dead, and dead cells were used to report the results. Three independent experiments (three independent biological replicates) were performed.

#### 2.3.5. Statistical Analysis

Samples were assayed in 3 replicates, and the results were provided as means ± SEM (standard error of the means). Statistical differences between experimental groups were evaluated using GraphPad Prism (GraphPad Prism version 9.3.0 for Windows, GraphPad Software, San Diego, CA, USA) and StatView software (version 5.0, SAS Institute Inc.), specifically the one-way ANOVA and Fisher’s tests. When the ANOVA was significant, means were compared using Tukey’s post hoc test. The *p*-values < 0.05 were considered statistically significant, whereas those < 0.1 showed a trend.

## 3. Results

### 3.1. Characterisation of Dried CP Extract

The HPLC analysis revealed a total phenolic compound concentration of 1.017 mg GAE/mL in the CP extract, comprising eighteen individual compounds representing different classes of phenolic acids and flavonoids (Table 1).

In the CP extract, flavan-3-ols are the predominant compounds, with epicatechin as the major component (40.0% of total phenolics), followed by catechin (16.2%) and epigallocatechin (15.0%). These three catechin derivatives collectively represented 71.3% of the total phenolic content, indicating their dominance in the extract profile. These compounds are recognised for their potent antioxidant activity and cardiovascular health benefits. Among hydroxycinnamic acids, chlorogenic acid was predominant (11.5%), followed by caffeic acid, ferulic acid, and methoxycinnamic acid. Chlorogenic acid is particularly valued for its antioxidant activity and beneficial effects on glucose metabolism. Hydroxybenzoic acids included vanillic acid, the most abundant (4.5%), followed by ellagic acid (4.0%), gallic acid (2.9%) (Table 1).

### 3.2. Evaluation of the Effects of CP Extract on IPEC-1 Monolayer Renewal Under In Vitro Conditions

Cell viability, proliferation and apoptosis were measured to evaluate epithelial renewal, an essential aspect of intestinal epithelia.

#### 3.2.1. The Effects of CP Extract on Cell Viability

**Figure 1:**
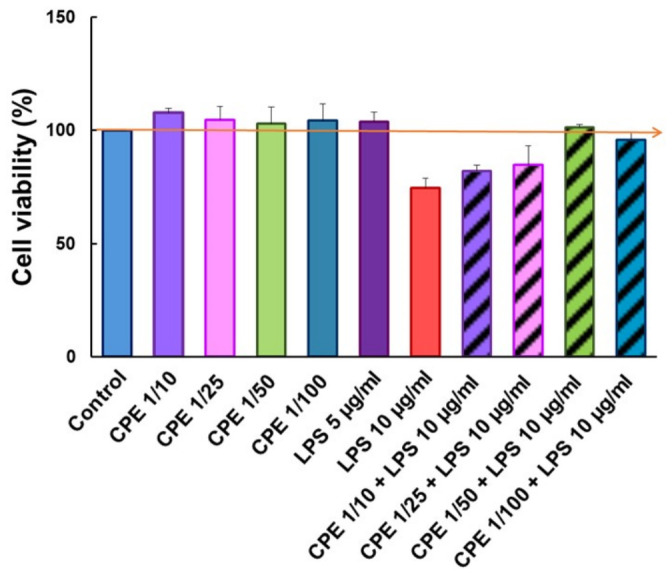
Effects of CP extract and LPS treatments on IPEC-1 cell viability. Cell viability was determined using the MTT assay at 24 h after incubation with CP extract and LPS. The results are representative of three independent experiments (three independent biological replicates) and are presented as percentages relative to control cells (untreated cells). Values are means, with standard errors represented by vertical bars.

To find the proper dilution of CPE to be used in cell culture experiments, we performed a previous MTT test using five dilutions of CPE (1/10, 1/25, 1/100 and 1/200) as well as two concentrations of LPS (5 and 10 μg/mL). Based on the results obtained using the MTT assay, we observed that CP extract had no cytotoxic effects on IPEC-1 cells, regardless of the dilution used (Figure 1). At the lowest concentration (dilution 1/10), a slight stimulation of cell proliferation was observed when compared to the control (*p* = 0.177), but the difference was not significant. The extract maintained cell viability at levels comparable to the control group, with values ranging from 98.5 ± 2.1% to 106.7 ± 3.8% (*p* > 0.05 for all comparisons, Figure 1). The results showed that LPS treatment at 5 μg/mL showed no significant cytotoxic effects compared to the control group (*p* = 0.972). By contrast, LPS treatment at 10 μg/mL reduced the cell viability to 69.3 ± 2.8%, a significant decrease of approximately 30% compared to the control group (*p* < 0.0001). Due to the pronounced effect on cell viability of 10 μg/mL LPS, we used this concentration in all further experiments. For further determinations of this experiment, we used concentrations of 1/50 and 1/100 of the CP extract, the cell viability in the case of CPE 1/50 + LPS and CPE 1/100 + LPS treatments being different from LPS and closer to the control group (over 95% viability in both conditions, *p* = 0.022 and *p* = 0.023 vs. LPS, respectively, Figure 1).

#### 3.2.2. The Effects of CP Extract on Cell Proliferation

**Figure 2:**
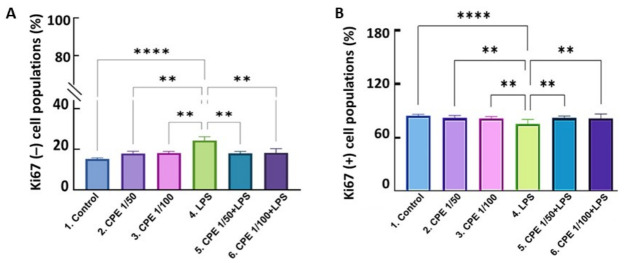
The effect of CP extract treatment on the Ki67 cell proliferation profile in IPEC-1 cells challenged with LPS. After the CPE and LPS treatments, cells were collected and stained for flow cytometry analysis as described in the Section 2. The results are representative of three independent experiments (three independent biological replicates) and are presented as means of cell populations not expressing Ki67 (**A**) or expressing (**B**) Ki67 marker, with standard errors represented by vertical bars. ** and **** = statistical significance, with *p* < 0.01 and *p* < 0.0001 respectively.

The graph in Figure 2A represents cells that do not express the proliferation marker protein Ki67 (−) and therefore cells that do not proliferate. It can be seen that cells challenged with LPS have the highest percentage of cells that do not express Ki-67 (−); therefore, they do not proliferate. Cells treated with polyphenolic extract of dried carrot pomace have fewer cells that did not proliferate; therefore, the percentage is lower. The percentage represents cells that do not express Ki-67.

Analysis of cell proliferation by quantification of Ki67 (+) IPEC1 cells showed that the percentage of proliferating cells was significantly reduced following LPS *E. coli* challenge (75.79%, *p* = 0.0001) compared to control (84.83%), as well as other treatments (Figure 2B and Figure S1). The results showed that the 1/50 and 1/100 dilutions of the CP extract successfully combat the harmful antiproliferative effect of *E. coli* LPS (82.07%, *p* = 0.003 and 81.80%, *p* = 0.004, respectively, Figure 2B and Figure S1).

#### 3.2.3. The Effects of CP Extract on Cell Apoptosis

**Figure 3:**
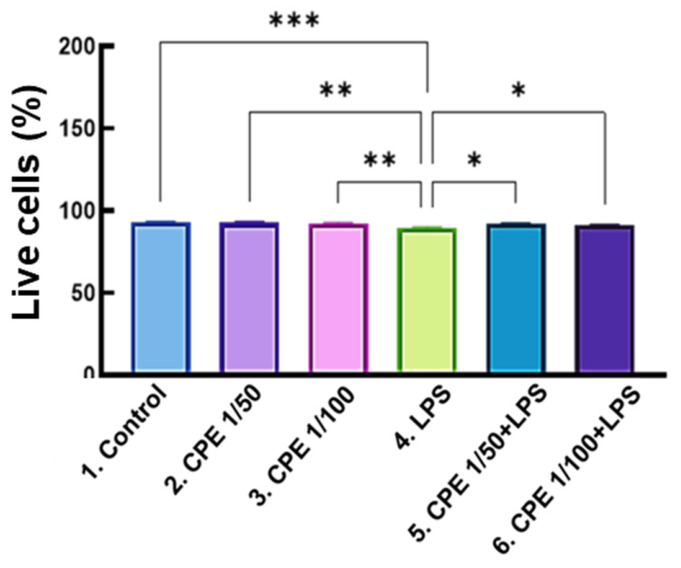
The effect of CP extract and LPS treatments on the percentage of live cells in IPEC-1 cell culture. After cell incubations with CPE and LPS treatments, cells were collected and stained for flow cytometry analysis as described in the Section 2. The results are representative of three independent experiments (three independent biological replicates) and are presented as means of the percentages reported relative to the control samples, with standard errors shown as vertical bars. *, ** and *** = statistical significance, with *p* < 0.05, *p* < 0.01 and *p* < 0.001 respectively. Y = cell viability (%).

The analysis of the percentage of total live cells (Figure 3) showed that the percentage of viable cells was significantly reduced when the cells were treated with *E. coli* LPS (89.31%, *p* = 0.0006) compared to untreated cells (Control, 93.03%). On the other hand, pre-treatment with 1/50 and 1/100 dilutions of CP extract alone led to an increase in this percentage compared to cells treated with LPS alone (91.63%, *p* = 0.010 and 91.13%, *p* = 0.035, respectively, Figure 3).

**Figure 4:**
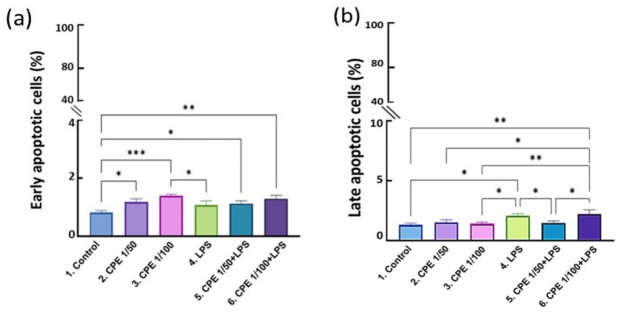
The effect of CP extract and LPS treatments on the percentage of early (**a**) and late (**b**) apoptotic IPEC-1 cells. After cell incubations with CPE and LPS treatments, cells were collected and stained for flow cytometry analysis as described in the Section 2. The results are representative of three independent experiments (three independent biological replicates) and are presented as means, with standard errors represented by vertical bars. *, ** and *** = statistical significance, with *p* < 0.05, *p* < 0.01 and *p* < 0.001 respectively.

Analysis of the early apoptotic phase showed that LPS induced cell apoptosis in 1.08% of cells compared to the control cells (0.83%, *p* = 0.096, Figure 4a). Surprisingly, CP at both 1/50 and 1/100 concentrations had a significant effect on the early apoptotic cells, increasing this percentage compared to the Control group (1.13%, *p* = 0.022 and 1.30%, *p* = 0.0005). All treatments had a negative impact compared to the control in the early apoptotic phase (Figure 4a).

In the late phase (late apoptotic) of the apoptotic process (Figure 4b), treatment with *E. coli* LPS had a significant influence on cell apoptosis, resulting in an increase in the percentage of cells in this phase (2.09%), both compared to the control (1.30%, *p* = 0.011) and to the other treatments. Dilutions of CP extract alone at 1/50 and 1/100 had no effect on the proportion of cells in the late stage of apoptosis compared to untreated cells (Control). When the cells were treated with 1/50 CP extract and then challenged with *E. coli* LPS, a significantly reduced proportion of apoptotic cells (1.48%, *p* = 0.0443) was observed compared to the cells treated only with LPS. The 1/100 dilution of the CP extract failed to counteract the effects of LPS, the percentage of late-stage apoptotic cells being similar to that in LPS-treated samples (2.21%), suggesting that a higher concentration of polyphenols from the CP extract is more effective in reducing late apoptosis.

**Figure 5:**
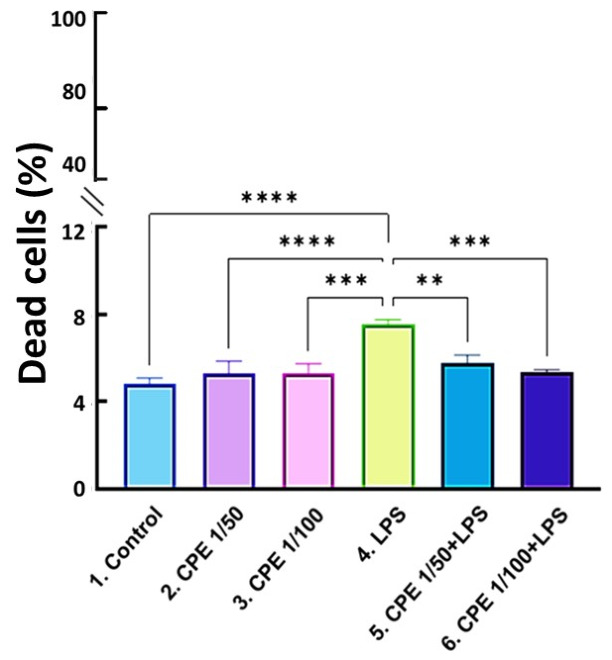
The effect of CP extract and LPS treatments on the percentage of dead cells in IPEC-1 cell culture. After cell incubations with CPE and LPS treatments, cells were collected and stained for flow cytometry analysis as described in the Section 2. The results are representative of three independent experiments (three independent biological replicates) and are presented as means, with standard errors represented by vertical bars. **, *** and **** = statistical significance, with *p* < 0.01, *p* < 0.001 and *p* < 0.0001 respectively.

The analysis of the total percentage of dead cells (Figure 5 and Figure S2) revealed that the LPS treatment leads to a significant increase in cell mortality percentages (7.53% dead cells *vs*. 4.48% in control cells, *p* < 0.0001). By contrast, the application of CP extract at dilutions of 1/50 and 1/100 before LPS treatment significantly decreased the percentages of dead cells, yielding 5.76% (*p* = 0.001) and 5.35% (*p* = 0.0001), respectively, when compared to LPS treatment alone.

### 3.3. In Vitro Assessment of CP Extract Effects on the Monolayer Integrity of IPEC-1 Cells Challenged with E. coli-LPS

#### 3.3.1. The Effects on Trans-Epithelial Electrical Resistance (TEER)

TEER measurements assessed barrier integrity under the action of *E. coli* LPS and the protective effects of the CP extract (Table 2). The control group showed stable TEER values throughout the experimental period, with minimal damage from +0.05% to +0.13% compared to T0 (baseline), indicating maintenance of barrier function. The treatments with CP extract alone at both concentrations showed a minimal impact on TEER, with a stable resistance comparable to control and values remaining within −0.54% to +0.20% of initial measurements. In contrast, LPS treatment caused severe barrier disruption, with a significant drop in TEER values at 24 h post-treatment; the values declined by −21.78%. This deterioration continued dramatically, reaching a −45.31% reduction at 48 h, and −53.47% at 72 h, indicating a substantial compromise of the epithelial barrier function. Pre-treatment with CP extracts significantly protected against LPS-induced dysfunction, with both dilutions maintaining TEER values near baseline (T0). The CPE (1/50) plus LPS maintained TEER values with minimal reductions of −0.19%, −0.11% and −0.43% at 24, 48 and 72 h, respectively, while CPE (1/100) plus LPS showed similar protective effects, with changes of +0.09%, −0.22% and −0.24% at the corresponding time points.

#### 3.3.2. The Effects on Cell Monolayer FD4 Permeability

The cells were cultured for 3 days until reaching confluence, then treated with polyphenolic extract for 4 h. After 4 h, the cells were challenged with LPS for another 24 h.

**Figure 6:**
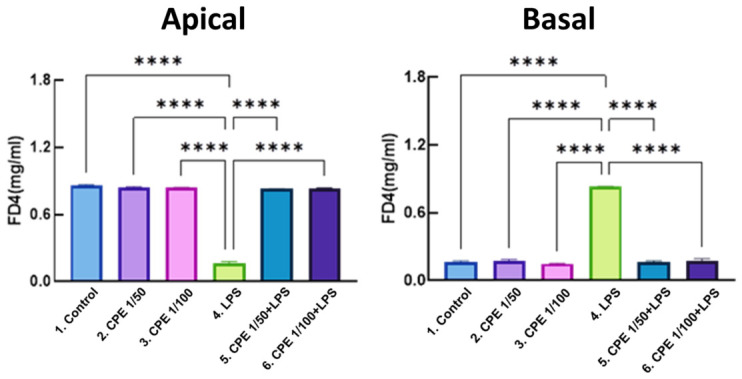
The effects of CP extract treatment on the monolayer permeability of IPEC-1cells challenged with LPS. After cell incubations with CPE and LPS treatments, FD4 was added to the apical side of the insert system as described in the Section 2. The results are representative of three independent experiments (three independent biological replicates) and are presented as means, with standard errors represented by vertical bars. **** = statistical significance, with *p* < 0.0001.

Under control conditions, the epithelial monolayer maintained intact barrier function (Figure 6), with FD4 predominantly being maintained in the apical compartment (0.855 ± 0.013 mg/mL) and minimal passage to the basal compartment (0.166 ± 0.016 mg/mL). The cells treated with the CP extract alone for 24 h did not significantly alter the barrier permeability compared to controls (*p* > 0.05). Exposure to LPS induced an obvious barrier disruption, as shown by decreased apical FD4 concentration (0.165 ± 0.013 mg/mL) and increased basal FD4 concentration (0.829 ± 0.016 mg/mL), indicating substantial paracellular leakage (*p* < 0.0001 *vs*. control for both compartments). Notably, pre-treatment with the CP extracts completely prevented LPS-induced barrier dysfunction. Cells treated with the polyphenolic extract at both concentrations plus LPS maintained FD4 distribution comparable to controls, with apical concentrations of 0.828 ± 0.013 mg/mL and 0.829 ± 0.013 mg/mL, respectively, and minimal basal passage (*p* < 0.0001 *vs*. LPS alone). No significant difference was observed between the two CP extract dilutions in their protective capacity.

#### 3.3.3. The Effects on Lactate Dehydrogenase (LDH) Release

**Figure 7:**
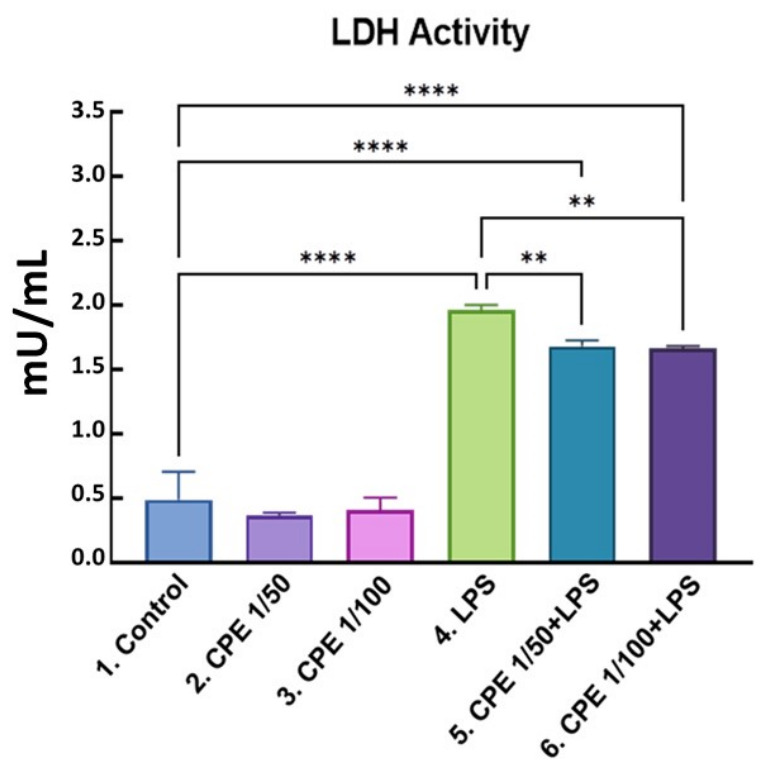
Evaluation of LDH activity in IPEC-1 cells in response to LPS stimulation and CP extract treatments. After cell incubations with CPE and LPS treatments, the LDH activity was determined in the cellular supernatant as described in the Section 2. The results are representative of three independent experiments (three independent biological replicates) and are presented as means, with standard errors represented by vertical bars. **, **** = statistical significance, with *p* < 0.01 and *p* < 0.0001 respectively.

Analysis of LDH activity in cell supernatants showed a significant difference in membrane damage induced by LPS when compared to control cells, with a *p*-value of less than 0.0001 (Figure 7). Furthermore, in cells that were pre-treated with CP before LPS challenge, a notable decrease in LDH concentration was detected at both the 1/50 dilution (*p* = 0.005) and the 1/100 dilution (*p* = 0.003) related to LPS-treated cells. These findings indicate that CP extract can mitigate the harmful effects of LPS on cell membranes, but it was not able to diminish the effect of LPS to the control level.

### 3.4. In Vitro Assessment of CP Extract Effect on the Functionality of IPEC-1 Cells Challenged with E. coli-LPS

To measure the potential of CP extracts to counteract the LPS-induced oxidative stress, we analysed the ROS and NO production of IPEC-1 cells as well as the morphology and membrane potential of mitochondria, one of the organelles where the largest number of free radicals (ROS) is generated, together with the energy necessary for cellular functioning.

#### 3.4.1. The Effects of Experimental Treatments on Oxidative Stress Protection

**Figure 8:**
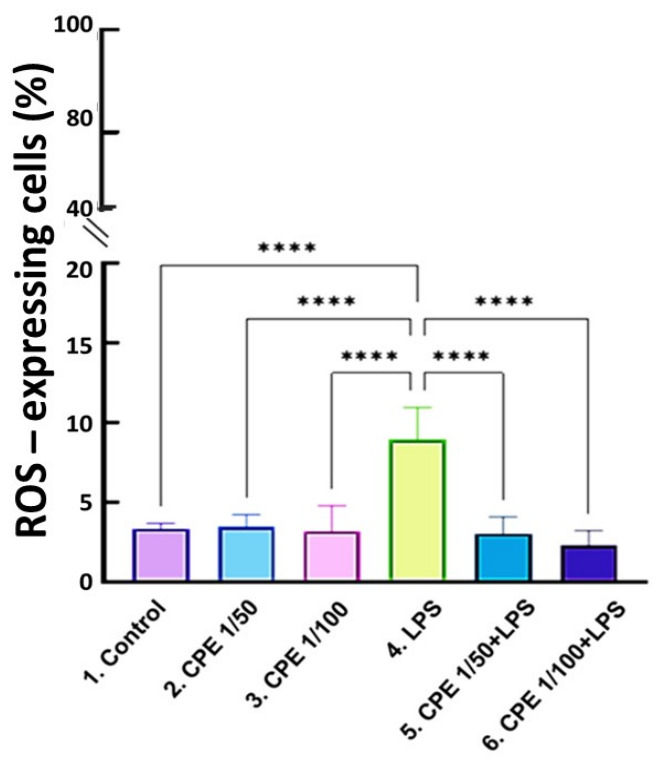
The effect of CP extract treatments on ROS expression in IPEC-1 cells challenged with LPS. After cell incubations with CPE and LPS treatments, cells were collected and stained for flow cytometry analysis as described in the Section 2. The results are representative of three independent experiments (three independent biological replicates) and are presented as means, with standard errors represented by vertical bars. **** = statistical significance, with *p* < 0.0001.

To assess the impact of CP extract on oxidative stress production, we quantified intracellular reactive oxygen species (ROS) levels and total nitric oxide (NO) production in cells treated with CP extract and challenged with LPS. Flow cytometry analysis demonstrated that the percentage of ROS (+) cell populations was low and stable under basal conditions in control cells, averaging 3.0% ROS (+) populations, and CPE treatments showed similar values (CPE 1/50 by 3.5% and CPE 1/100 by 3.2%), indicating minimal oxidative stress (Figure 8 and Figure S3). The cells challenged with LPS increased the ROS production up to 9.0% (*p* < 0.0001 *vs*. control), nearly a 3-fold increase. The pre-treatment with CP extract and then challenge with LPS significantly mitigated the effects of the ROS production in a dose-dependent manner, reducing ROS (+) cells to 3.0% in the 1/50 dilution challenged with LPS and 2.5% in the 1/100 dilution challenged with LPS (both treatments with *p* < 0.0001).

**Figure 9:**
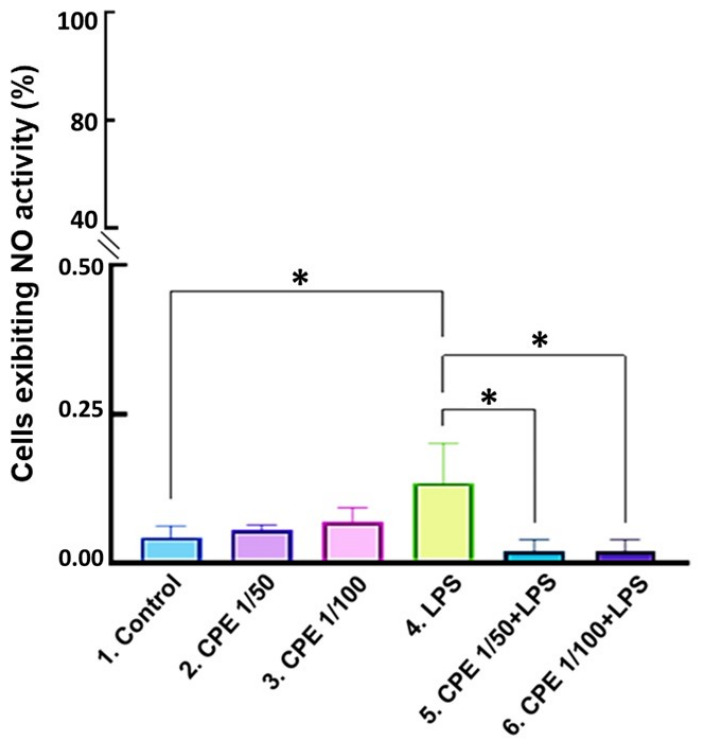
The effect of treatments with CP extract on the nitrosative stress in the IPEC-1 cells challenged with LPS. After cell incubations with CPE and LPS treatments, cells were collected and stained for flow cytometry analysis as described in the Section 2. The results represent the average of three independent experiments (three independent biological replicates) and are presented as means, with standard errors. * = statistical significance, with *p* < 0.05.

In the cells challenged with LPS, the percentage of cells with NO activity registered a 3.5-fold increase when compared to control (untreated) cells (*p* = 0.048 *vs*. control, Figure 9 and Figure S4), while the pre-treatment with CP extract and then challenge with LPS lowered the percentage of cells with NO (+) with 7-fold (CPE 1/50 + LPS: *p* = 0.005 and CPE 1/100 + LPS: *p* = 0.007 *vs*. LPS, Figure 9 and Figure S4).

#### 3.4.2. The Effects on Mitochondrial Morphology (MitoLite Assay)

**Figure 10:**
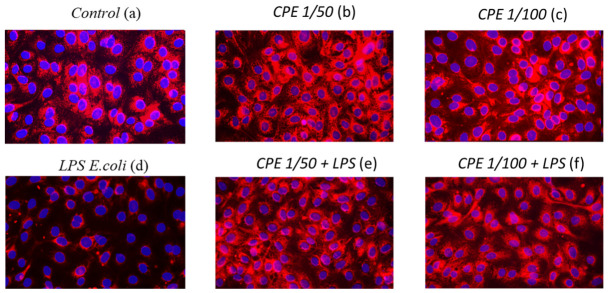
The effects on mitochondrial morphology in IPEC-1 cells treated with CP extract and challenged with LPS. After cell incubation with CPE and LPS treatments, the MitoLite assay was performed as described in the Section 2. The images are representative of three independent experiments (three independent biological replicates). (**a**) Control cells; (**b**) cells treated with CPE 1/50; (**c**) cells treated with CPE 1/100; (**d**) cells treated with LPS; (**e**) cells pre-treated with CPE 1/50 and after that challenged with LPS; (**f**) cells pre-treated with CPE 1/100 and after that challenged with LPS.

Cells treated with CP extract alone (1/50 and 1/100 dilutions) showed a mitochondrial staining pattern comparable to untreated control cells (Figure 10a–c). While this treatment did not affect mitochondrial morphology, the cells challenged with LPS alone were visibly altered (Figure 10d). The observed changes are consistent with mitochondrial stress, the altered fluorescence distribution patterns suggesting mitochondrial fragmentation. Cells pre-treated with CPE and then challenged with LPS showed dose-dependent protective effects on mitochondrial integrity, with the highest effect at dilution 1/50, as demonstrated by well-preserved mitochondrial morphology and fluorescence intensity comparable to the control (Figure 10e,f).

**Figure 11:**
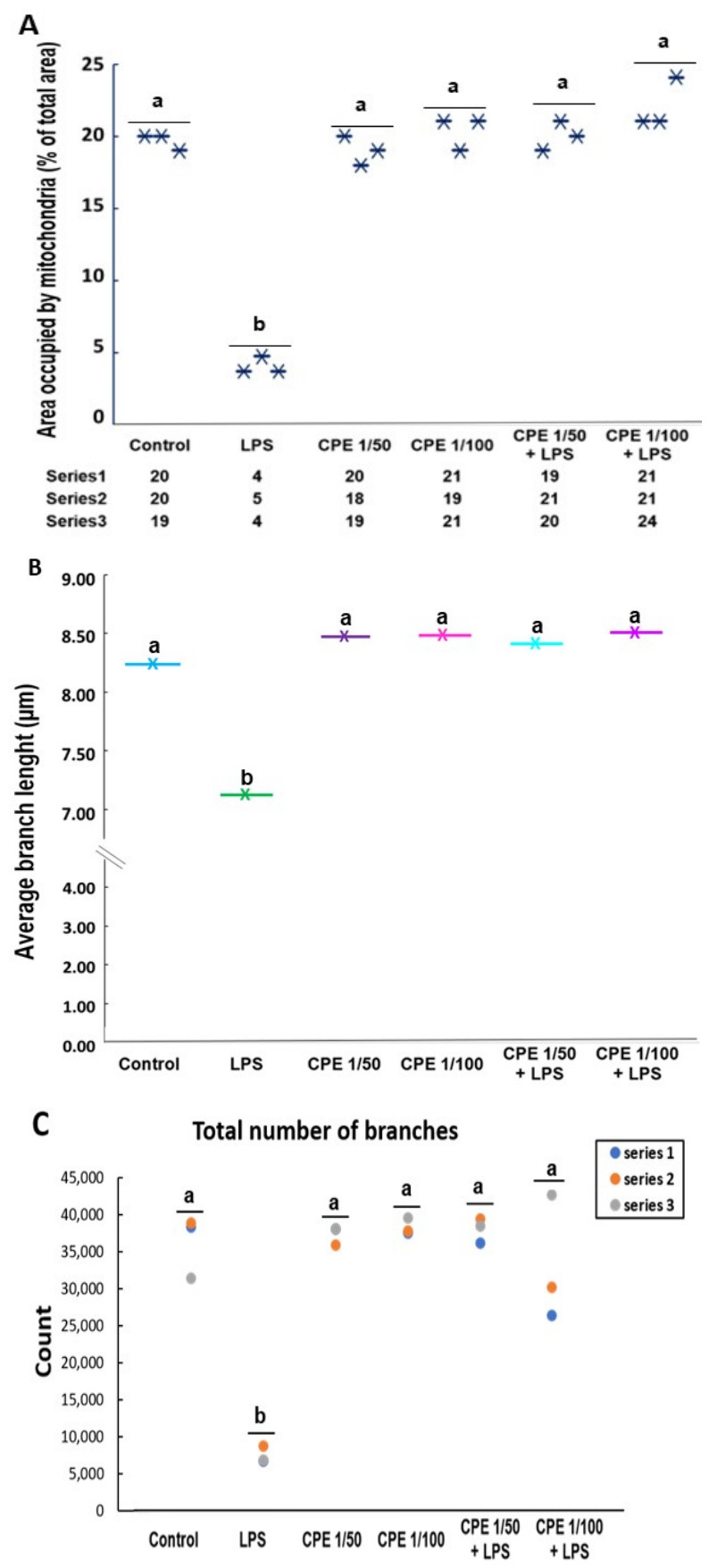
The effects of the cellular treatments on mitochondrial fragmentation. Summary statistics of mitochondrial network analysis in CPE- and LPS-treated IPEC1 cells: (**A**) area occupied by mitochondria, (**B**) average branch length, (**C**) total number of branches. Points represent individual series values for three independent experiments (three independent biological replicates). ^a^, ^b^ Different superscript letters indicate significant differences between cellular treatments (*p* < 0.050).

The quantitative analysis of MitoLite Red FX600-stained IPEC1 cells demonstrated that LPS-challenged cells exhibited a significant reduction in the total area occupied by mitochondria when compared to control cells (Figure 11A, *p* < 0.001 *vs*. control), as well as a decreased mean mitochondrial branch length and number (Figure 11B,C, *p* < 0.001 *vs*. control). This structural aspect of mitochondria in LPS-challenged cells shows a collapsed mitochondrial network, in accordance with MitoLite images. Cells pre-treated with CPE before LPS challenge preserve their mitochondrial features, showing morphological characteristics similar to those of control cells (Figure 11A–C).

#### 3.4.3. The Effects on the Cell’s Mitochondrial Membrane Potential

**Figure 12:**
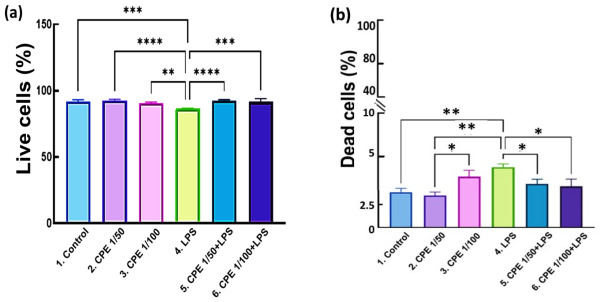
The effect of treatments with CP extract on the MitoPotential profile of live (**a**) and dead (**b**) IPEC-1 cells challenged with LPS. After cell incubations with CPE and LPS treatments, cells were collected and stained for flow cytometry analysis as described in the Section 2. The results are representative of three independent experiments (three independent biological replicates) and are presented as means, with standard errors represented by vertical bars. *, **, *** and **** = statistical significance, with *p* < 0.05, *p* < 0.01, *p* < 0.001 and *p* < 0.0001 respectively.

The LPS treatment significantly reduced the percentage of live cells to 87% (*p* = 0.0003) and increased the percentage of dead cells to 5.2% (*p* = 0.004) compared to control cells (Figure 12a,b). CP extract pre-treatment provided significant protection; the treatment with 1/50 dilution followed by LPS challenge restored the percentage of live cells to 93% (*p* < 0.0001 *vs*. LPS) and reduced the percentage of dead cells to 3.8% (*p =* 0.036). Similarly, the treatment with CP extract 1/100 dilution combined with LPS maintained 92% of living cells (*p* = 0.0003 *vs*. LPS) with 3.5% dead cells (*p* = 0.019) (Figure 12a,b).

**Figure 13:**
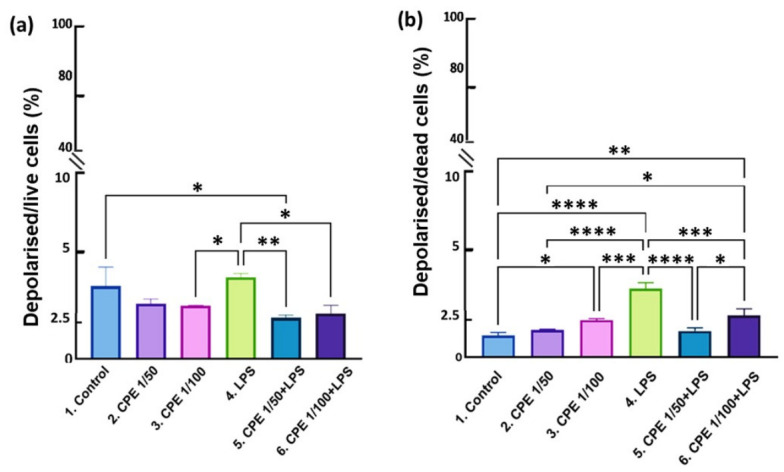
The effect of treatments with CP extract on Mitopotential profile of depolarised/dead (**a**) and depolarised/live (**b**) IPEC-1 cells challenged with LPS. After cell incubations with CPE and LPS treatments, cells were collected and stained for flow cytometry analysis as described in the Section 2. The results are representative of three independent experiments (three independent biological replicates) and are presented as means, with standard errors represented by vertical bars. *, **, *** and **** = statistical significance, with *p* < 0.05, *p* < 0.01, *p* < 0.001 and *p* < 0.0001 respectively.

Analysis of depolarised/live cell populations showed that LPS treatment increased the proportion of depolarised live cells to 4% and 4.5% compared to the control. CP extract treatments alone showed a reduction compared to control (by 3.0% at 1/50, *p* = 0.187 and by 2.8% at 1/100, *p* = 0.154), but with no statistical significance. The CP extract pre-treatments of the cells, plus further LPS challenge, significantly reduced the depolarised/live cells population compared to LPS (by 2.2% at 1/50 dilution plus LPS, *p* = 0.007 and by 2.5% at 1/100 dilution plus LPS, *p* = 0.013), indicating protection of mitochondrial integrity in viable cells (Figure 13a).

In the case of depolarised/dead cells, control cells showed minimal depolarisation (1.2%), whereas the LPS treatment induced a visible increase to 3.7% (*p* < 0.0001), indicating mitochondrial dysfunction (Figure 13b and Figure S5). CP extract (1/50) alone did not affect mitochondrial depolarisation, being close to control (*p* = 087 *vs*. control), and a similar behaviour was observed when cells were treated with CP extract (1/50) and challenged with LPS. CP extract (dilution 1/100), alone or in combination with LPS, also resulted in a lower percentage of depolarised cells compared to LPS treatment alone. However, the addition of CP extract (dilution 1/100) alone or in combination with LPS increased the percentage of depolarised/dead cells above the control (Figure 13b).

#### 3.4.4. The Effects on Pro-Inflammatory Cytokine Expression

To assess how the function of intestinal epithelium to synthesise effector molecules is affected by LPS and CP extracts, we measured the expression of genes coding for pro-inflammatory cytokines (*IL-6*, *TNFα* and *IL-1β*), in cells treated with CP extract alone (1/50 and 1/100 dilution), *E. coli*-LPS, or their combinations (Table 3). Gene expression was analysed using qPCR and normalised to control levels, with results expressed as fold change. CP extracts alone led to a modest increase in *IL-6* expression by 35% (CPE 1/50, *p* = 0.049 *vs*. control) and by 18% (CPE 1/100, *p* = 0.304 *vs*. control). As expected, the cells challenged with LPS induced the highest *IL-6* expression, with a 48% increase over control (*p* = 0.014). The interaction of CP extracts with LPS for 24 h significantly mitigated the LPS-induced *IL-6* response (22.3% and 40% reduction in the case of 1/50 and 1/100 dilutions, respectively, Table 3).

*TNF-α* gene expression increased by 49% after the challenge of the cells with LPS (*p* = 0.044). The interaction of CP extracts with LPS after 4 h of incubation showed a reducing effect by 50% for the CPE 1/50 plus LPS challenge (*p* = 0.005), and by 56% for the CPE 1/100 plus LPS challenge (*p* = 0.002) compared to LPS. *TNF-α* expression was not modified by the CP extracts alone compared to control (*p* = 0.259 and *p* = 0.917, respectively, Table 3).

The LPS challenge of the cells showed a 60% increase in *IL-1β* gene expression (*p* = 0.029 *vs*. control). The treatment of the cells with CP extracts before adding LPS counteracted the increasing effect produced by LPS compared to the control. The CP extracts treatments alone did not alter the *IL-1β* expression compared to control (CPE 1/50: *p* = 0.938, CPE 1/100: *p* = 0.621).

#### 3.4.5. The Effects on Signalling Pathway Molecules

**Figure 14:**
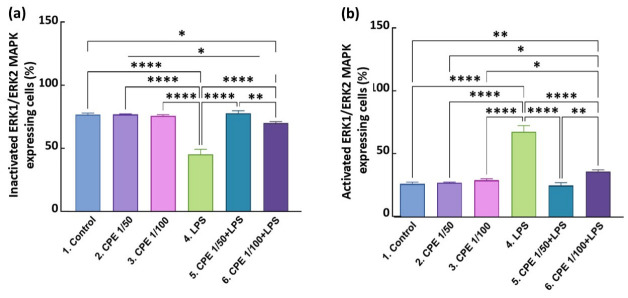
The effect of treatments with CP extract on inactivated (**a**) and activated (**b**) ERK1/ERK2 MAPK’s expression, in IPEC-1 cells challenged with LPS. After cell incubations with CPE and LPS treatments, cells were collected and stained for flow cytometry analysis as described in the Section 2. The results are representative of three independent experiments (three independent biological replicates) and are presented as means, with standard errors represented by vertical bars. *, ** and **** = statistical significance, with *p* < 0.05, *p* < 0.01 and *p* < 0.0001 respectively.

To investigate the mechanism of action of LPS and CP extracts, we determined by flow cytometry the percentage of cells expressing inactivated and phosphorylated/activated Mitogen-Activated Protein Kinases (MAPK3-ERK1 and MAPK1-ERK2), key signalling molecules involved in inflammation. The treatment with CP extracts alone exhibited minimal effects on cells expressing both inactivated and activated ERK1/ERK2 MAPKs (Figure 14a,b and Figure S6). By contrast, the percentage of cells expressing inactivated ERK1/ERK2 MAPKs was significantly reduced in LPS-challenged IPEC1 cells (−40.0% reduction, *p* = 0.001 *vs*. control, Figure 14a and Figure S6). Also, the expression of activated ERK1/ERK2 MAPKs was strongly induced by LPS compared to control cells (+156.3% increase, *p* < 0.0001 *vs*. control, Figure 14b and Figure S6). The pre-treatment with 1/50 dilution of CPE plus LPS significantly increased the expression of inactivated ERK1/ERK2 MAPKs on IPEC1 cells (by 58.2%, *p* < 0.0001 *vs*. LPS) and reduced the percentage of cell populations expressing activated ERK1/ERK2 MAPKs by 62.9% (*p* < 0.0001 *vs*. LPS), fully restoring it to baseline levels (*p* = 0.318 *vs*. control and *p* = 0.697 *vs*. control, respectively). Similarly, the combined treatment between CPE 1/100 dilution and LPS increased the percentage of cells expressing inactivated ERK1/ERK2 MAPKs by 36.8% (*p* < 0.001 *vs*. LPS), diminishing the percentage of cells with overexpressed activated ERK1/ERK2 MAPKs by 46.5% (*p* < 0.001 *vs*. LPS), maintaining levels toward the control values (*p* = 0.123 and *p* = 0.052, respectively).

## 4. Discussions

This study shows that CP extract possesses significant protective properties against LPS-induced intestinal epithelial damage in IPEC-1 cells. Our analyses revealed multiple beneficial effects, including protection of epithelial renewal, epithelial barrier integrity, mitochondrial protection, antioxidant activity and selective immunomodulation of pro-inflammatory cytokines.

The biochemical characterisation of the CP extract showed a total phenolic concentration of 1.017 mg/mL was found, with flavan-3-ols (epicatechin, catechin, epigallocatechin) representing over 71% of the total phenolic content [26,27]. The presence of flavan-3-ols in the CP extract is particularly relevant due to their known anti-inflammatory and barrier-protective effects in intestinal epithelial cells [28]. In their study, Zhang and Vasconcelos showed that epicatechin, a known flavan-ols reduces inflammatory markers like *TNF-α*, *IL-6* and nitric oxide (NO) while inhibiting *NF-kB* activation; it increases the proliferating cell nuclear antigen (PCNA) and epidermal growth factor (*EGF*) expression, therefore stimulating epithelial cell proliferation and restoration [29]. Furthermore, catechins flavanols have been associated with the prevention and treatment of intestinal inflammation through direct and indirect antioxidant effects and the regulation of immune cell infiltration and proliferation [30]. The extract also contains other polyphenols such as chlorogenic acid, known for its several health effects on antioxidant activity, inflammation, glucose regulation, etc. It has been demonstrated that chlorogenic acid improves intestinal barrier function by suppressing mucosal inflammation and enhancing antioxidant capacity [31,32,33].

The results on cell viability showed that the CP extract maintained cell viability to levels comparable to the controls, with no cytotoxic effects observed at any tested dilution. The safety profile is important for the future use of carrot-derived by-products in animal feed. The *E. coli* LPS treatment at 10 μg/mL significantly reduced cell viability by up to 69.3%, a fact that is consistent with its well-known cytotoxic effect on intestinal epithelial cells [34]. while CP extract had a protective effect on the cell proliferation against LPS-induced damage, the results showed that the percentage of Ki67 positive cells significantly reduced after the LPS treatment, returning towards the control level (75.79% LPS *vs*. 84.83% in control). The pre-treatment with CP extract at both dilutions 1/50 and 1/100 counteracted the effects induced by LPS, restoring the proliferation rates up to 82.07% and 81.80%, respectively. Our results are similar to other studies on plant polyphenols, where the protocatechuic acid, quercetin and luteolin have been shown to attenuate ETEC-induced inflammation and injury in IPEC-1 cells and maintain the cell proliferation capacity [35,36]. The LPS treatment showed in the analysis of apoptosis that the percentage of dead cells increased to 7.53% vs. 4.48% in controls, and in late apoptotic cells to 2.09% vs. 1.30%. Pre-treatment of cells with the CP extracts for 4 h significantly mitigated the effects of LPS by reducing the percentage of dead cells to 5.76% (1/50 dilution) and 5.35% (1/100 dilution).

Significant findings were obtained in our study concerning the protection of CP extract on the intestinal epithelial barrier by analysing the trans-epithelial electrical resistance (TEER), paracellular permeability (FD4) and LDH release assay. The results showed that the cells pre-treated with CP extract maintained the TEER values near the control baseline after the challenge with LPS. The FD4 assay provides additional information to TEER measurements, being often used to study the gastrointestinal paracellular permeability [37]. These results are in agreement with a protective effect of polyphenols against barrier dysfunction by upregulation of tight junction proteins, as previously shown [38,39,40].

The results from Huang’s study on IPEC J2 cells suggest that the apple polyphenols promote intestinal antioxidant capacity and the barrier function via the Nrf2/Keap1 signalling pathway [38]. Nunes showed in his study on HT-29 cells that red wine extract reinforced the intestinal barrier under inflammatory conditions by preserving the tight junctions in intestinal epithelial cells, enhancing the key proteins like occludin and ZO-1 and reducing inflammatory markers [39]. Polyphenols are known for their ability to modulate key regulators of barrier function, such as AMPK and MLCK, and protect the tight junction proteins from oxidative damage [40]. Even if the FD4 assay has some limitations [41], when it is correlated with TEER measurements and tight junction protein expression analysis, it provides a comprehensive assessment of the barrier integrity [42]. The FD4 and LDH assays further confirmed the barrier protective effects of CP extract in the pre-treated cells, significantly reducing the membrane damage induced by LPS. Although the LDH levels remained elevated compared to control, the results indicate that the CP extract can partially mitigate the cytotoxic effect of LPS on the cell membranes. It is possible that either the two concentrations cannot fully repair membrane damage and reduce LDH to values close to control, or that higher concentrations are needed or that a longer time of action of the extract on the cells is needed. We assume that in zootechnical practice, piglets receive a diet with carrot pomace for a longer period (at least one month until the transition to another category, that of growing pigs) and that it is possible that lower concentrations also have an effect.

It was demonstrated that polyphenolic compounds can modulate mitochondrial function and prevent the caspase activation cascades involved in apoptosis [6]. Our study showed that CP extract offers significant, dose-dependent protection against the cellular stress induced by LPS on mitochondrial function. The fluorescence microscopy revealed that LPS caused mitochondrial fragmentation and disrupted the fluorescence patterns, while the pre-treated cells with CP extract maintained a normal mitochondrial morphology. These results were confirmed by the quantitative analysis of the mitochondria-stained images, demonstrating that CPE pre-treatment of IPEC1 cells preserves their mitochondrial features and prevents the decrease in mean mitochondrial branch length and number and the collapsed mitochondrial network induced by LPS.

The flow cytometry assay confirmed that LPS reduced the live cell population, and the CP extract restored the live cell population after they were challenged with LPS. These results line up with the findings of Carrasco-Pozo et al.’s study, who revealed similar mitochondrial protection from apple peel polyphenols on indomethacin-challenged CaCO_2_ cells [43]. These effects are likely due to the polyphenols’ free radical scavenging properties, which help in the prevention of oxidative damage and maintain the electron transport chain functions. The results, which are dose-dependent, imply that higher concentrations of polyphenols can offer much bigger mitochondrial protection, a fact that is consistent with other studies in cell models (Caco2, HepG2 cells) [43,44].

As a known powerful inflammatory stimulus through TLR4 activation, LPS induced a significant increase in pro-inflammatory *IL-6* (48% increase relative to control) and *TNF-α* (49% increase relative to control) cytokine gene expression. Pre-treatment of cells with CP extract significantly mitigated this inflammatory response, reducing *IL-6* expression by 40% compared to LPS alone and *TNF-α* expression by 50 to 56%. These findings align with other studies on carrot-derived compounds, which demonstrated that purple carrot anthocyanins can downregulate the mRNA expression of pro-inflammatory interleukins *IL-1β* (by 91%) and *IL-6* (by 69%) in the co-culture of intestinal CaCO_2_ and macrophage RAW264.7 cells challenged with LPS [45]. Similarly, the polyphenols from other varieties of carrots, like for example the black carrot, have been shown to downregulate the secretion of pro-inflammatory markers, including IL-8, monocyte chemoattractant protein 1, vascular endothelial growth factor and intercellular adhesion molecule 1 after gastrointestinal digestion under inflammatory conditions [46]. In our study, the modulation of *IL-6* and *TNF-α*, with no significant differences between treatments, suggests that CP extract may have a stronger effect on some pro-inflammatory pathways than others.

The MAPK signalling cascade that includes ERK1/2, p38 and JNK pathways has an important role in the inflammatory responses induced by LPS [47]. In our study, the ERK1/ERK2 MAPK expression increased dramatically in the cells challenged with LPS (156.3% above control). By contrast, this activation was reduced by the pre-treatment of the cells with CP extract, the 1/50 dilution reducing the activated ERK1/ERK2 MAPK expression by 62.9%, and that of 1/100 by 46.5%, with returning levels to baseline. It seems that higher concentrations are more effective in combating the effect of LPS. These findings are consistent with other studies that show that the activation of the MAPK pathway is a characteristic of inflammatory responses induced by LPS and contributes to the upregulation of pro-inflammatory cytokines and other mediators [48]. Many studies *in vitro* and *in vivo* have shown that natural plant extracts that are rich in polyphenolic compounds have anti-inflammatory effects by inhibiting the MAPK signalling cascade and reducing the cellular inflammatory responses [49,50,51]. Besides polyphenols, carrot pomace also contains other compounds with biological activity. For example, Yung et al. (2020) showed in their study that β-carotene, a precursor of vitamin A, reduced the intestinal inflammation induced by LPS in both rats and IEC-6 cells, with the modulation of autophagy and by regulating the JAK2/STAT3 and MAPK signalling pathways [52]. These examples suggest that the bioactive compounds from carrots can be used as modulators of inflammation by targeting MAPK regulation.

Carrot compounds have proven not only anti-inflammatory but also antioxidant properties. The antioxidant capacity of the CP extract was demonstrated by the significant reduction of reactive oxygen species and nitric oxide production. As expected, the LPS treatment increased the percentage of ROS (+) cells (9% vs. 3% in control), and it was completely normalised by the pre-treatment of the cells with CP extract at both dilutions (3% and 2.5%, respectively). These findings are consistent with other studies showing antioxidant properties of bioactive compounds of CP [53]. For example, the β-carotene found in CP is not only an anti-inflammatory but also an antioxidant compound with activity against oxidative stress and lipid peroxidation. It was shown that β-carotene reduced the marker of lipid peroxidation and increased the GSH levels in the mice colon with ulcerative colitis induced by DSS. In the same study, β-carotene also decreased the oxidative DNA damage in the colon of mice, demonstrating its antioxidant property [54]. Also, chlorogenic acid is a phenolic acid that can be found in carrots and their by-products, which showed an antioxidant effect by lowering extracellular H_2_O_2_ and intracellular ROS, reducing the oxidative stress in IPEC-J2 cells challenged with LPS [33]. Other studies demonstrated that chlorogenic acid inhibits the NO production of inflammation-related factors and inflammatory cytokines in a dose-dependent manner and attenuates the inflammatory state induced by LPS with the modulation of *NF-kB* signalling cascades [55,56]. Also, in our study the production of NO decreased although from a statistical point of view the reduction was not significant.

In conclusion, this study demonstrates that CP extract has a protective effect against the damage produced by LPS on the intestinal epithelial cells through multiple mechanisms that can operate at both cellular, molecular and biochemical levels. The protective effect at the level of intestinal epithelial cells involves the inhibition of ERK1/ERK2 MAPK expression, the reduction of pro-inflammatory cytokine expression, and the preservation of mitochondrial functionality and antioxidant activity. Further *in vivo* studies are needed to validate these *in vitro* results and to establish the dietary concentration/rate of inclusion of carrot by-products to achieve the maximal positive effects.

## Figures and Tables

**Table 1:** Phenolic compound composition of CP extract.

Compound	mg/mL	Compound	mg/mL
Epicatechin	0.407	Vanillic acid	0.046
Catechin	0.165	Ellagic acid	0.041
Epigallocatechin	0.153	Gallic acid	0.029
Chlorogenic acid	0.117	Rutin	0.020
Caffeic acid	0.011	Ferulic acid	0.006
Syringic acid	0.005	Methoxy cinnamic acid	0.005
Protocatechuic acid	0.003	p-Coumaric acid	0.002
3-Hydroxybenzoic acid	0.002	Trans-cinnamic acid	0.002
Kaempferol	0.001	Resveratrol	n.d.
Total phenolic content			1.017

**Table 2:** TEER measurements (Ω) across different treatment groups and time points.

Time	Experimental Treatments *					
	Control	CPE 1/50	CPE 1/100	LPS	LPS+ CPE 1/50	LPS+ CPE 1/100
T0	13,113.25 ^a^	13,103.00 ^a^	13,093.00 ^a^	13,051.00 ^a^	13,090.25 ^a^	13,070.75 ^a^
24 h	13,129.75 ^a^	13,092.50 ^a^	13,098.75 ^a^	10,208.75 ^b^	13,065.00 ^a^	13,083.00 ^a^
48 h	13,124.25 ^a^	13,068.50 ^a^	13,119.00 ^a^	7139.00 ^b^	13,076.25 ^a^	13,042.00 ^a^
72 h	13,120.25 ^a^	13,032.50 ^a^	13,107.50 ^a^	6073.00 ^b^	13,034.00 ^a^	13,039.75 ^a^

* After cell incubations with CPE and LPS treatments, the trans-epithelial electric resistance was measured at moment 0, after 24 h, 48 h and 72 h, respectively, as described in the Section 2. The results are representative of three independent experiments and are presented as means. ^a^, ^b^ = mean values within a row with unlike superscript letters were significantly different (*p* < 0.05).

**Table 3:** The effects of treatments on the expression of genes coding for pro-inflammatory cytokines.

Gene	Experimental Treatments *					
	Control	CPE 1/50	CPE 1/100	LPS	CPE1/50 + LPS	CPE1/100 + LPS
*IL-6*	1.00 ^b^	1.35 ^b^	1.18 ^b^	1.48 ^a^	1.15 ^b^	0.89 ^b^
*TNFα*	1.00 ^b^	0.74 ^b^	1.00 ^b^	1.49 ^a^	0.75 ^b^	0.66 ^b^
*IL-1β*	1.00 ^b^	0.98 ^b^	1.13 ^b^	1.60 ^a^	1.34 ^b^	1.18 ^b^

* After cell incubations with CPE and LPS treatments, the genes coding for *IL-6*, *TNF-α* and *IL-1β* pro-inflammatory cytokines were determined by qPCR, as described in the Section 2. The results are expressed as fold changes (Fc) relative to the control levels and are representative of three independent experiments (three independent biological replicates) and are presented as means. ^a^, ^b^ Different superscript letters indicate significant differences between cellular treatments (*p* < 0.050).

## Data Availability

The datasets used and/or analysed during the current study are available from the corresponding author on reasonable request.

## Supplementary Materials

The following supporting information can be downloaded at: https://www.mdpi.com/article/10.3390/antiox15070847/s1, **Supplementary Table S1**. The sequences of primers used for qPCR amplification, **Supplementary Figure S1**. Representative histogram of Ki67 proliferation profile in IPEC-1 cells treated with CP extract and challenged with LPS, **Supplementary Figure S2**. Representative dot plots sorted by flow cytometry of Annexin V-stained populations in IPEC-1 cells treated with CP extract and LPS, **Supplementary Figure S3**. Representative histogram of ROS population profile in IPEC-1 cells treated with CP extract and challenged with LPS, **Supplementary Figure S4**. Representative dot plots sorted by flow cytometry of Nitric Oxide profile in IPEC-1 cells treated with CP extract and LPS, **Supplementary Figure S5**. Representative dot plots sorted by flow cytometry of Mitopotential cell profile in IPEC-1 cells treated with CP extract and challenged with LPS, **Supplementary Figure S6**. Representative dot plots sorted by flow cytometry analysis of ERK21/ERK2 MAPK expression in IPEC-1 cells treated with CP extract and challenged with LPS.
