# Supplementary material for: Assessing the In Vitro Effects of Carrot Pomace Extract on Intestinal Epithelium Integrity and Functions
Source: Antioxidants (Basel). 2026 Jul 4;15(7):847. doi: 10.3390/antiox15070847 (PMC13403770; doi:10.3390/antiox15070847)

Supplementary Table S1. The sequences of primers used for qPCR amplification.

Gene	Description	Accession no.	Primer source	Primer sequence (5`→3`)	Orientation	Amplicon length (bp)	References
TNF-α	Tumour necrosis factor-α	NM_214022	Pig	ACTGCACTTCGAGGTTATCGG	forward	118	[1]
				GGCGACGGGCTTATCTGA	reverse		
IL-6	Interleukin-6	NM_214399	Pig	GGCAAAAGGGAAAGAATCCAG	forward	87	[1]
				CGTTCTGTGACTGCAGCTTATCC	reverse		
IL-1β	Interleukin-1β	NM_214055	Pig	ATGCTGAAGGCTCTCCACCTC	forward	89	[2]
				TTGTTGCTATCATCTCCTTGCAC	reverse		
B-2 mg	β 2 microglobulin	NM_213978	Pig	TTCTACCTTCTGGTCCACACTGA	Forward	162	[3]
				TCATCCAACCCAGATGCA	Reverse		
GAPDH	Glyceraldehyde-3-Phosphate Dehydrogenase	NM_001206359.1	Pig	ACTCACTCTTCTACCTTTGATGCT	Forward	100	[3]
				TGTTGCTGTAGCCAAATTCA	Reverse		
CypA	Cyclophilin A	NM_214353.1	Pig	CCCACCGTCTTCTTCGACAT	Forward	92	[3]
				TCTGCTGTCTTTGGAACTTTGTCT	Reverse		
ACTB	β-actin	NM_213978.1	Pig	GGACTTCGAGCAGGAGATGG	Forward	230	[3]
				GCACCGTGTTTGCGTAGAGG	Reverse		
HPRT-1	Hypoxanthine phosphoribosyl transferase 1	NM_001032376.2	Pig	TGGAAAGAATGTCTTGATTGTTGAAG	Forward	93	[3]
				ATCTTTGGATTATGCTGCTTGACC	Reverse		
RPL 32	Ribosomal Protein L32	NM_001001636	Pig	TGCTCTCAGACCCCTTGTGAAG	Forward	106	[3]

1. Grenier, B.; A. P. Bracarense; H. E. Schwartz; C. Trumel; A. M. Cossalter; G. Schatzmayr; M. Kolf-Clauw; W. D. Moll; I. P. Oswald. The low intestinal and hepatic toxicity of hydrolyzed fumonisin B_1 correlates with its inability to alter the metabolism of sphingolipids. *Biochemical pharmacology* **2012**, *83*, 1465-1473, doi:10.1016/j.bcp.2012.02.007.

2. Von Der Hardt, Katharina; Michael Andreas Kandler; Ludger Fink; Ellen Schoof; Jörg Dötsch; Olga Brandenstein; Rainer Maria Bohle; Wolfgang Rascher. High Frequency Oscillatory Ventilation Suppresses Inflammatory Response in Lung Tissue and Microdissected Alveolar Macrophages in Surfactant Depleted Piglets. *Pediatric Research* **2004**, *55*, 339-346, doi:10.1203/01.PDR.0000106802.55721.8A.
3. Pistol G. C., Marin D. E., Bulgaru V. C., Anghel A. C., Saracila M., Vlassa M., Filip M., Taranu I. Grape seed meal by-product is able to counteract oxidative stress induced by lipopolysaccharide and dextran sulphate in IPEC cells and piglets after weaning. *PloS one* **2023**, *18*, e0283607, doi:10.1371/journal.pone.0283607.

Supplementary Figure S1. Representative histogram of Ki67 proliferation profile in IPEC-1 cells treated with CP extract and challenged with LPS.

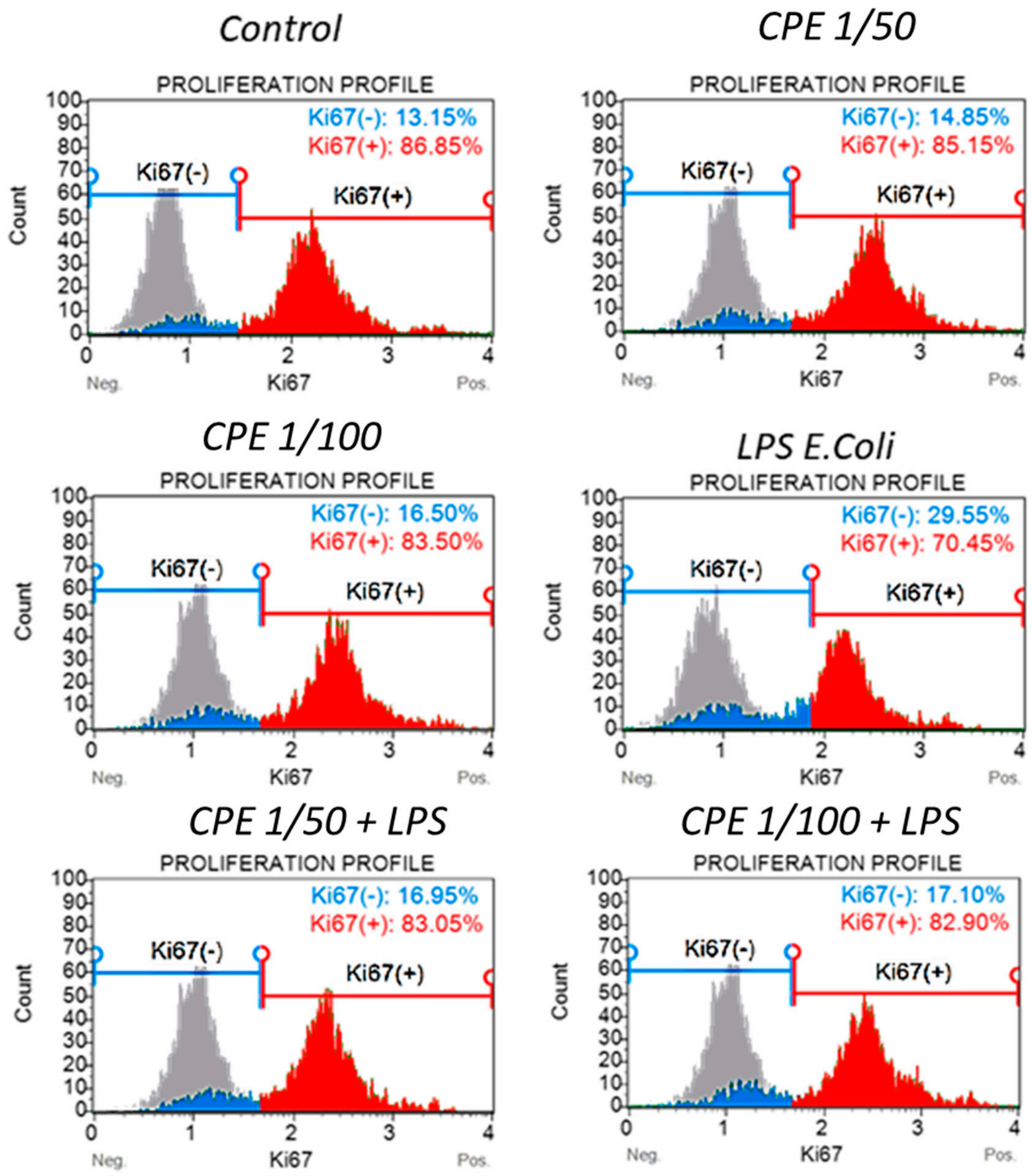

Supplementary Figure S2. Representative dot plots sorted by flow cytometry of Annexin V-stained populations in IPEC-1 cells treated with CP extract and LPS.

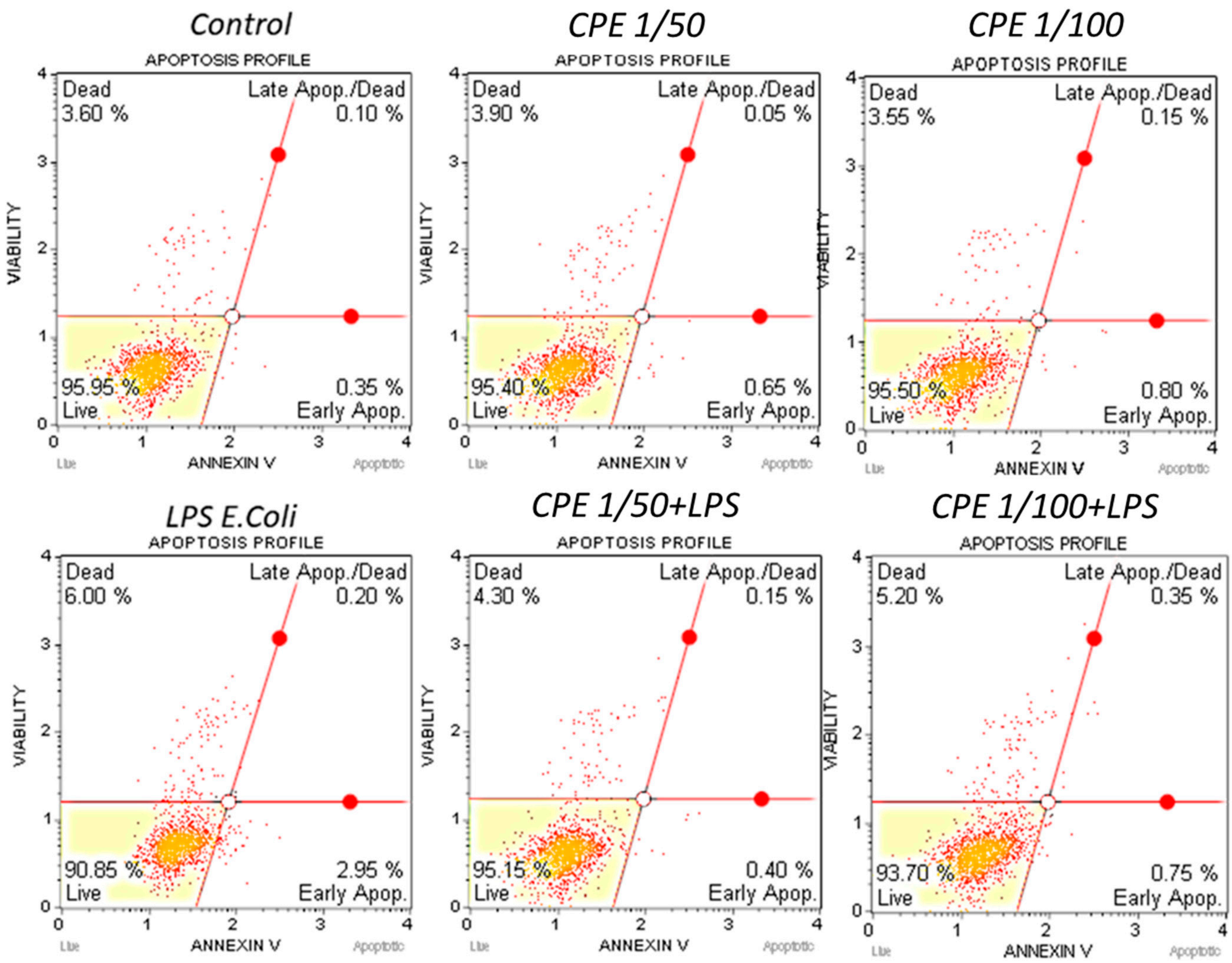

Supplementary Figure S3. Representative histogram of ROS population profile in IPEC-1 cells treated with CP extract and challenged with LPS.

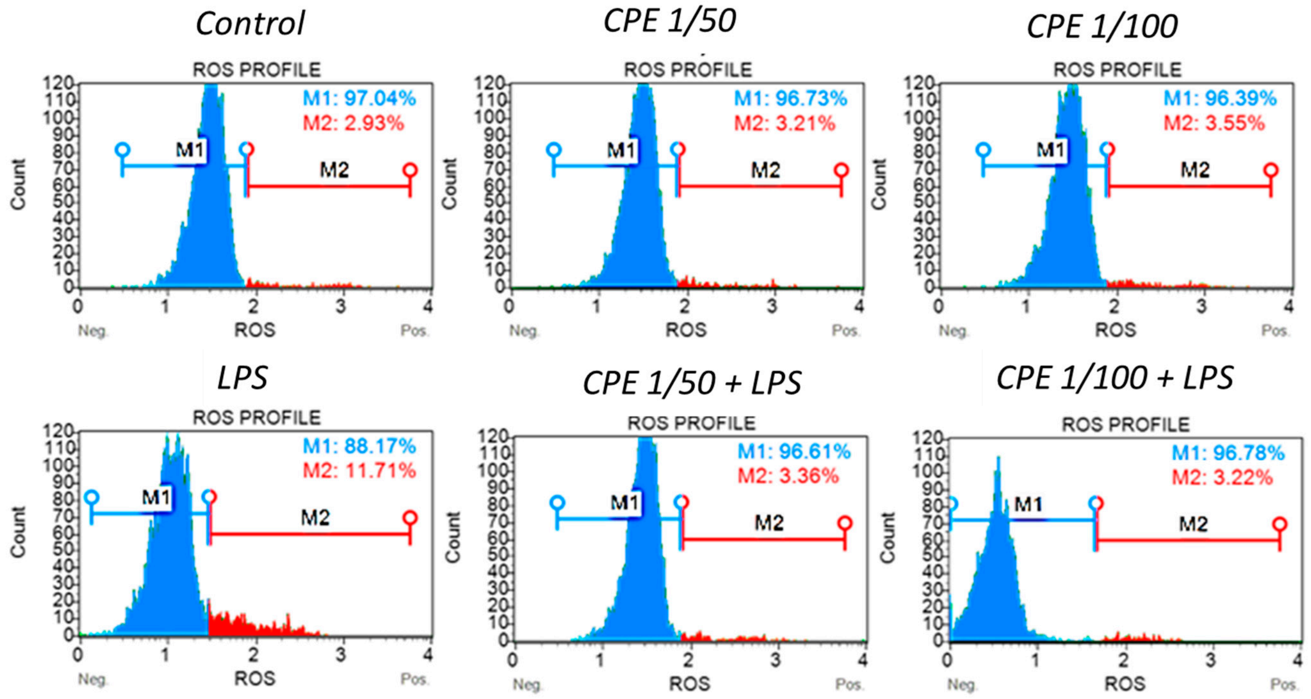

Supplementary Figure S4. Representative dot plots sorted by flow cytometry of Nitric Oxide profile in IPEC-1 cells treated with CP extract and LPS.

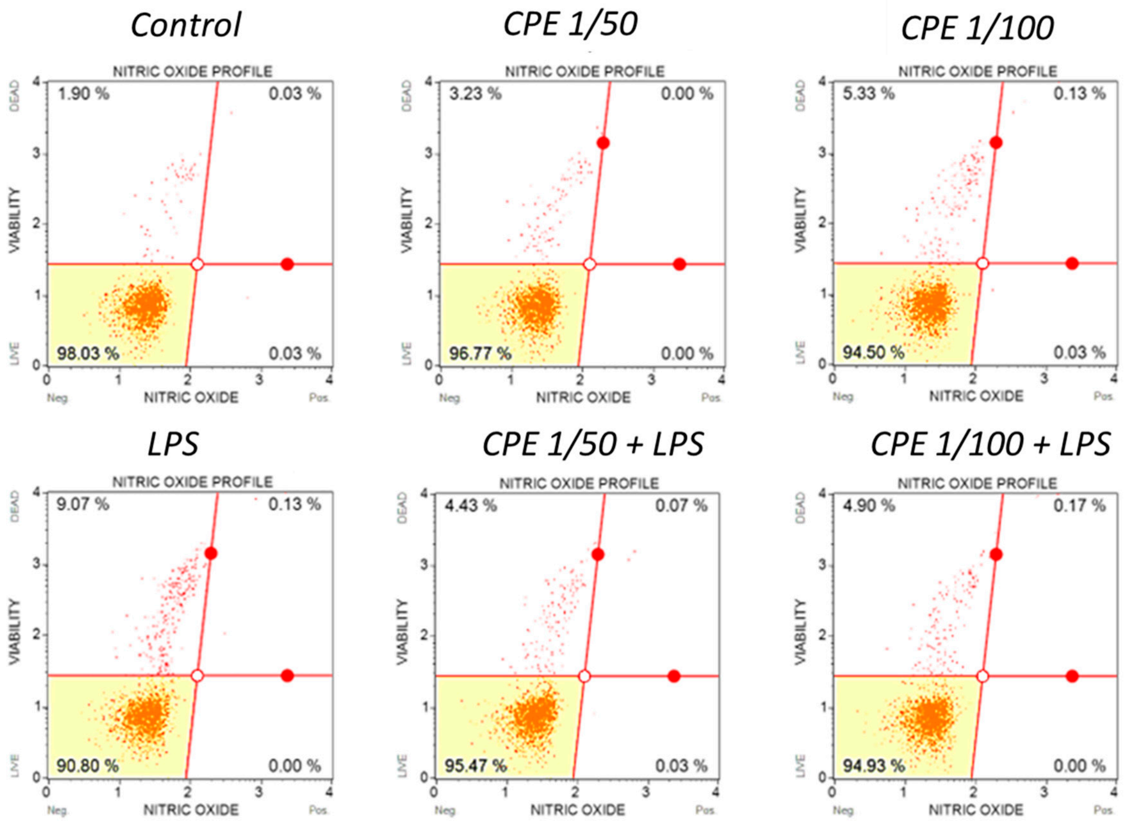

Supplementary Figure S5. Representative dot plots sorted by flow cytometry of Mitopotential cell profile in IPEC-1 cells treated with CP extract and challenged with LPS.

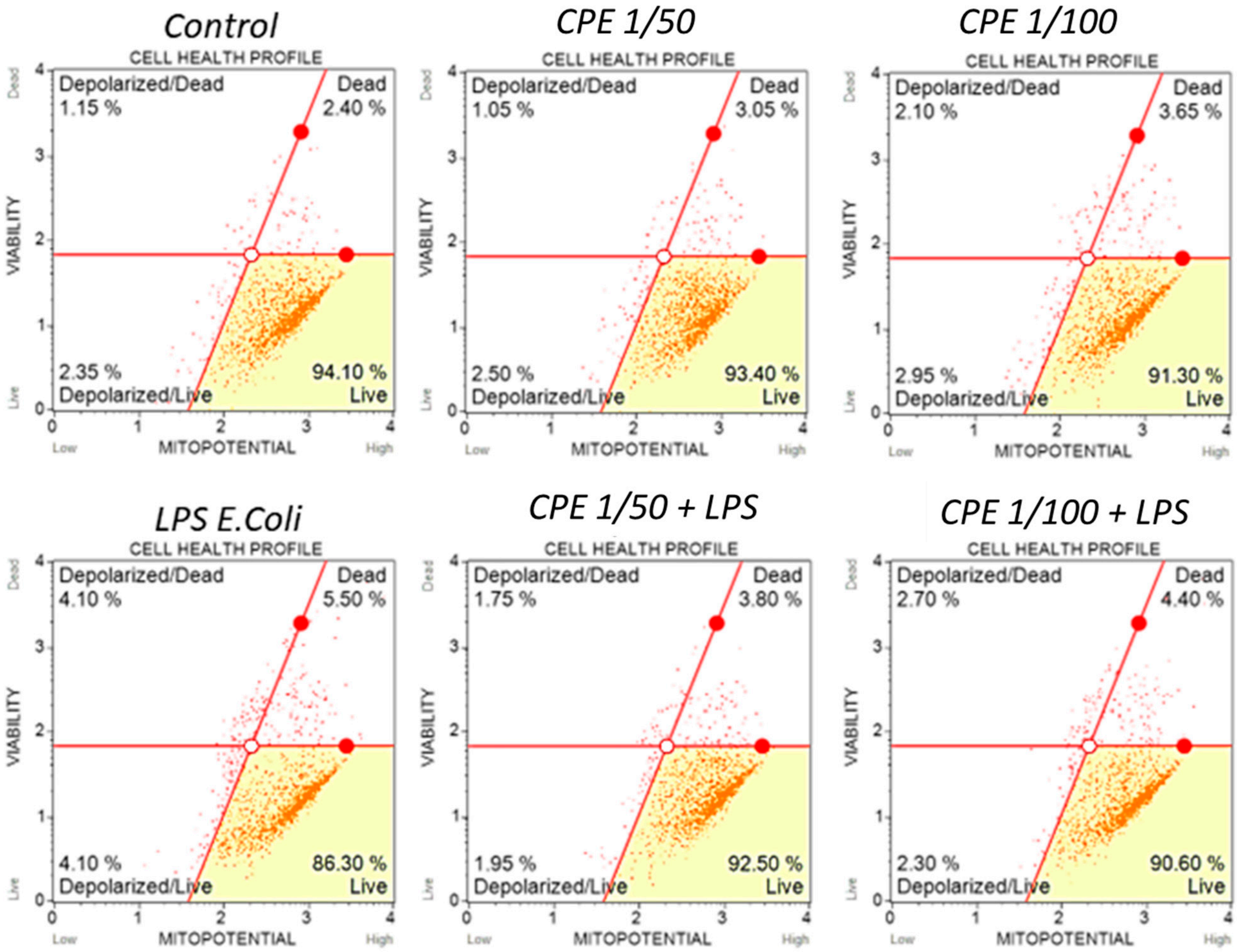

Supplementary Figure S6. Representative dot plots sorted by flow cytometry analysis of ERK21/ERK2 MAPK expression in IPEC-1 cells treated with CP extract and challenged with LPS.

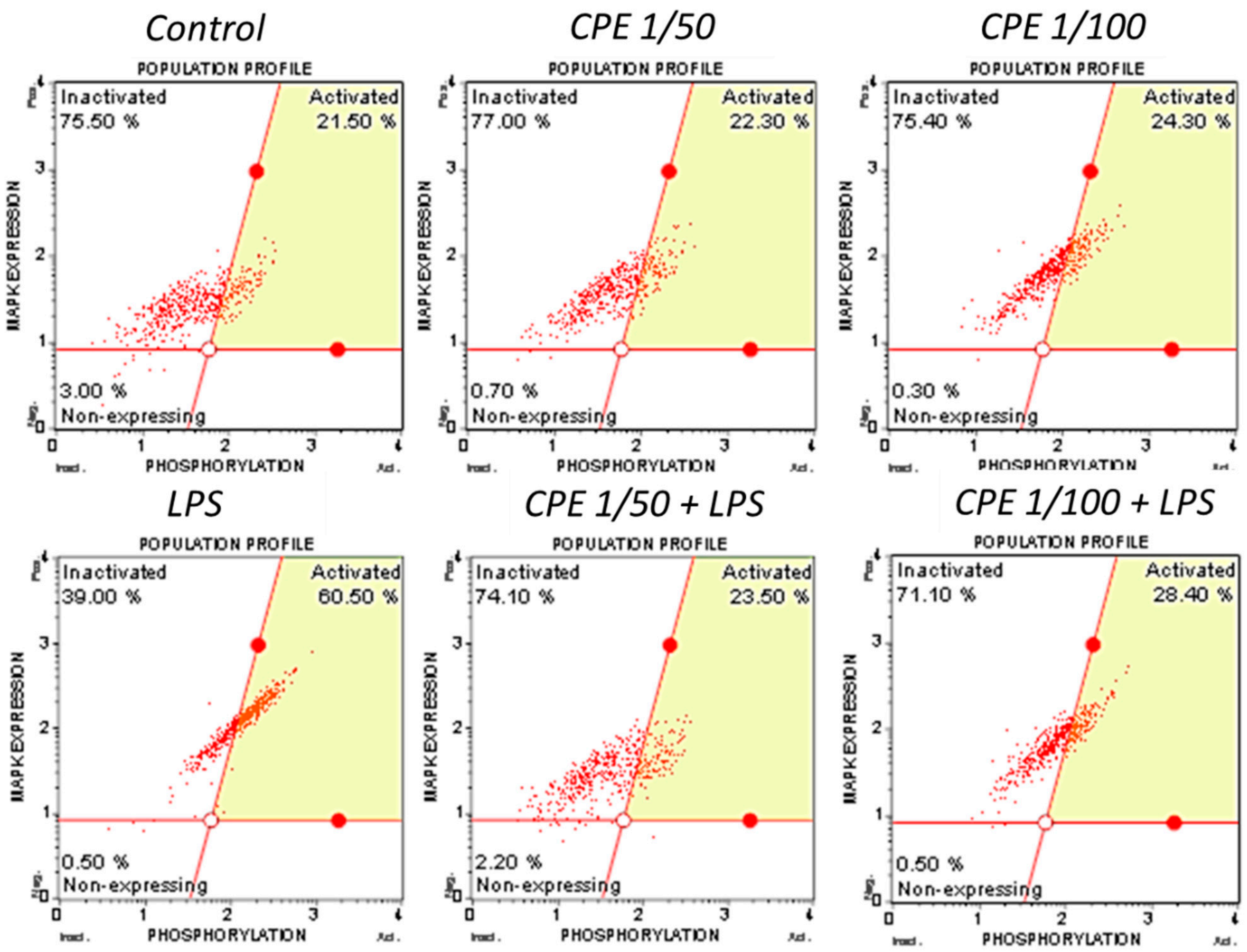

Supplement: Supplementary file 1 [file antioxidants-15-00847-s001.zip › antioxidants-4345313-supplementary.pdf]
